# Ependyma: a new target for autoantibodies in neuromyelitis optica?

**DOI:** 10.1093/braincomms/fcac307

**Published:** 2022-11-30

**Authors:** Maxime Bigotte, Marie Gimenez, Antoine Gavoille, Adamantia Deligiannopoulou, Aseel El Hajj, Severine Croze, Abdelghafar Goumaidi, Gael Malleret, Paul Salin, Pascale Giraudon, Anne Ruiz, Romain Marignier

**Affiliations:** FORGETTING Team—Lyon Neuroscience Research Center, INSERM U1028, CNRS UMR 5292, Claude Bernard Lyon 1 University, 69675 Bron, France; FORGETTING Team—Lyon Neuroscience Research Center, INSERM U1028, CNRS UMR 5292, Claude Bernard Lyon 1 University, 69675 Bron, France; Service de neurologie, sclérose en plaques, pathologies de la myéline et neuroinflammation, Hôpital Neurologique Pierre Wertheimer, Hospices Civils de Lyon, 69500 Bron, France; Service de Biostatistique-Bioinformatique, Hospices Civils de Lyon, 69495 Pierre-Bénitem, France; FORGETTING Team—Lyon Neuroscience Research Center, INSERM U1028, CNRS UMR 5292, Claude Bernard Lyon 1 University, 69675 Bron, France; FORGETTING Team—Lyon Neuroscience Research Center, INSERM U1028, CNRS UMR 5292, Claude Bernard Lyon 1 University, 69675 Bron, France; Profilexpert, Genomic and Microgenomic Service, Claude Bernard Lyon 1 University, SFR santé LYON-EST, UCBL-INSERM US 7-CNRS UMS 3453, 69008 Lyon, France; Viroscan3D SAS, 69008 Lyon, France; FORGETTING Team—Lyon Neuroscience Research Center, INSERM U1028, CNRS UMR 5292, Claude Bernard Lyon 1 University, 69675 Bron, France; FORGETTING Team—Lyon Neuroscience Research Center, INSERM U1028, CNRS UMR 5292, Claude Bernard Lyon 1 University, 69675 Bron, France; FORGETTING Team—Lyon Neuroscience Research Center, INSERM U1028, CNRS UMR 5292, Claude Bernard Lyon 1 University, 69675 Bron, France; FORGETTING Team—Lyon Neuroscience Research Center, INSERM U1028, CNRS UMR 5292, Claude Bernard Lyon 1 University, 69675 Bron, France; FORGETTING Team—Lyon Neuroscience Research Center, INSERM U1028, CNRS UMR 5292, Claude Bernard Lyon 1 University, 69675 Bron, France; Service de neurologie, sclérose en plaques, pathologies de la myéline et neuroinflammation, Hôpital Neurologique Pierre Wertheimer, Hospices Civils de Lyon, 69500 Bron, France; Centre de référence des maladies inflammatoires rares du cerveau et de la moelle (MIRCEM), 69500 Bron, France

**Keywords:** ependymal cell, neuromyelitis optica, aquaporin-4, cilia, sub-ventricular zone

## Abstract

Neuromyelitis optica (NMO) is an autoimmune demyelinating disease of the central nervous system characterized by the presence of autoantibodies (called NMO-IgG) targeting aquaporin-4. Aquaporin-4 is expressed at the perivascular foot processes of astrocytes, in the glia limitans, but also at the ependyma. Most studies have focused on studying the pathogenicity of NMO-IgG on astrocytes, and NMO is now considered an astrocytopathy. However, periependymal lesions are observed in NMO suggesting that ependymal cells could also be targeted by NMO-IgG. Ependymal cells regulate CSF-parenchyma molecular exchanges and CSF flow, and are a niche for sub-ventricular neural stem cells. Our aim was to examine the effect of antibodies from NMO patients on ependymal cells. We exposed two models, i.e. primary cultures of rat ependymal cells and explant cultures of rat lateral ventricular wall whole mounts, to purified IgG of NMO patients (NMO-IgG) for 24 hours. We then evaluated the treatment effect using immunolabelling, functional assays, ependymal flow analysis and bulk RNA sequencing. For each experiment, the effects were compared with those of purified IgG from a healthy donors and non-treated cells. We found that: (i) NMO-IgG induced aquaporin-4 agglomeration at the surface of ependymal cells and induced cell enlargement in comparison to controls. In parallel, it induced an increase in gap junction connexin-43 plaque size; (ii) NMO-IgG altered the orientation of ciliary basal bodies and functionally impaired cilia motility; (iii) NMO-IgG activated the proliferation of sub-ventricular neural stem cells; (iv) treatment with NMO-IgG up-regulated the expression of pro-inflammatory cytokines and chemokines in the transcriptomic analysis. Our study showed that NMO-IgG can trigger an early and specific reactive phenotype in ependymal cells, with functional alterations of intercellular communication and cilia, activation of sub-ventricular stem cell proliferation and the secretion of pro-inflammatory cytokines. These findings suggest a key role for ependymal cells in the early phase of NMO lesion formation.

## Teaser

Bigotte *et al*. report that autoantibodies from neuromyelitis optica patients can alter ependymal cells’ morphology and functions using an in vitro and an ex vivo model of ependyma. This study enlarges our understanding of neuromyelitis optica pathogenesis and highlights the pathogenicity of autoantibodies present in the cerebrospinal fluid.

## Graphical Abstract

**Graphical abstract:**
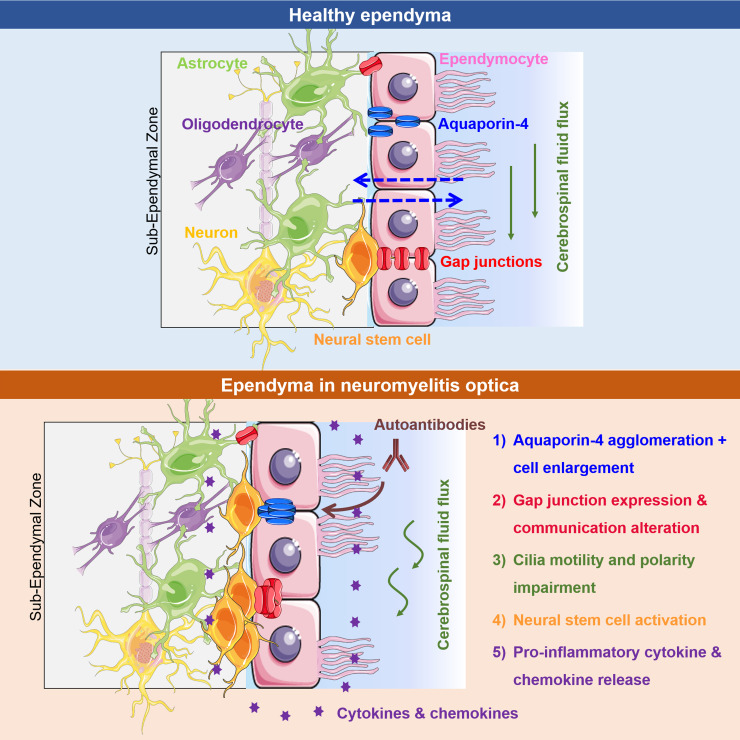

## Introduction

Neuromyelitis optica (NMO) is a rare neuroinflammatory demyelinating disease of the central nervous system, mostly characterized by optic nerve and spinal cord lesions.^1^ NMO was previously considered a sub-type of multiple sclerosis until highly specific autoantibodies [NMO-immunoglobulin G (IgG)] were found in a large majority of patients.^2^ NMO-IgG targets aquaporin-4 (AQP4), the main water channel of the brain expressed on astrocytic basal foot processes, the sub-arachnoid glia limitans and ependyma.^3^ Since then, the pathogenic role of NMO-IgG has been evaluated, leading to the consensus that NMO is a primary astrocytopathy.^4,5^ Histopathological evaluation revealed that six different types of astrocytic lesions can be found concomitantly or depending on the clinical evolution, suggesting that multiple pathogenic mechanisms can support the link between NMO-IgG–induced astrocytopathy and tissue injury leading to demyelination and axonal loss.^6,7^ These mechanisms include direct and bystander complement-dependent cytotoxicity, and bystander antibody-dependent cell-mediated cytotoxicity.^8^ In parallel, NMO-IgG can also be directly pathogenic by inducing pro-inflammatory cytokine secretion, extracellular glutamate release, or glial communication via gap junction alteration.^9–12^ Thus, the pathogenic role of NMO-IgG on astrocytes is well documented. However, little is known about the effects of NMO-IgG on ependymocytes, which also express AQP4.

**Table 1:** Primary and secondary antibodies used in immunolabelling

Primary antibodies				
Target	Host	Dilution	Reference	
AQP-4	Rabbit	1/100	Millipore AB2218	
β-catenin	Rabbit	1/250	Sigma c2206	
β-catenin	Mouse	1/500	BD Transduction Laboratories 610153	
Connexin 43	Mouse	1/500	BD Transduction Laboratories 610062	
FoxJ1	Mouse	1/500	eBioscience (Fisher) 14-9965-82	
γ-tubulin	Mouse	1/500	Abcam ab11316	
Ki67	Rabbit	1/500	Abcam ab15580	
**Secondary antibodies**				
**Target**	**Fluorochrome**	**Host**	**Reference**	**Dilution**
Rabbit IgG	AF 488	Goat	Life Technologies A-11034	1/1000
Mouse IgG	AF 488	Goat	Life Technologies A-11029	1/1000
Rabbit IgG	AF 555	Goat	Life Technologies A-21429	1/1000
Mouse IgG	AF 555	Goat	Life Technologies A-21424	1/1000
Mouse IgG	AF 647	Goat	Life Technologies A-21236	1/1000
Human IgG	Biotin-SP conjugated	Donkey	Jackson 709-065-149	1/500
Biotin	AF 488	Streptavidin	Jackson 016-540-084	1/1000
Mouse IgG	Biotin-SP conjugated	Goat	Jackson 115-065-008	1/1000
Biotin	AF 647	Streptavidin	Jackson 016-600-084	1/1000

Here are the references and the working dilutions used for immunolabelling ependymal cells from primary cultures and rat lateral ventricle whole mounts.

**Table 2:** Primers for the qRT-PCR

Gene name	Primers
TNF	5′ CTC AAG CCC TGG TAT GAG CC 3′, 5′ CGG GCA GGT CTA CTT TGG AG 3′
IL-6	5′ GTC AAC TCC ATC TGC CCT TCA 3′, 5′ CTT CCA GCC AGT TGC CTT CT 3′
IL-10	5′ TTG AAC CAC CCG GCA TCT AC 3′, 5′ TAA CGG GAG CAA CTC CTT GG 3′
IL-33	5′- GTG CCC TGA GCA CAT ACA AC-3′, 5′ CCA TCC ACA CCG TCT CCT GA 3′
CXCL1	5′ TGC ACC CAA ACC GAA GTC AT 3′, 5′ ACT TGG GGA CAC CCT TTA GC 3′
CXCL3	5′ CAG TGC TAA GAG ACG GGA ATG 3′, 5′ TCC TTA GGT ATG AAA GGT CTG CT 3′
CXCL9	5′ TGT GGA GTT CGA GGA ACC CT 3′, 5′ ACC CTT GCT GAA TCT GGG TC 3′
CXCL13	5′ CCT CCA GGC CAC GGT ATT C 3′, 5′ TCA GTT TTG GGG CAG CCA TT 3′
CCL2	5′ TGT CTC AGC CAG ATG CAG TT 3′, 5′ TCG GCT GAG TAA CCC TAG TA 3′
CCL6	5′ CAA GCC AGG CAT CAT CTT TGT 3′, 5′ TCC CAG ATC TTG GGC CTA GC 3′
CCL12	5′ CCG GGA AGC TGT GAT CTT CA 3′, 5′ CTA TCG CAC TGT CCA TGG GG 3′
CCL19	5′ GTC TTC CTC CAA GAG CAA AGG 3′, 5′ CAC TCA CGT TCA CAC CGA CT 3′

Here are the primers used for the quantification of cytokine and chemokine transcripts identified in the RNASeq experiment.

Ependyma is an epithelial layer composed of cuboid multiciliated cells called ependymocytes that line all the inner cavities of the brain filled with cerebrospinal fluid (CSF). At the interface between CSF and the central nervous system parenchyma, ependymal cells regulate important homeostatic functions such as molecular exchanges, CSF propulsion via synchronous ciliary beating and sub-ventricular neural stem cell (SV-NSC) survival and proliferation.^13,14^ These functions, mediated by cilia are in part supported by gap junction communication.^15^ Structural and functional alterations of ependymal cells have been observed in neuroinflammatory models such as stroke or experimental autoimmune encephalomyelitis.^16,17^ In these models, ependymal cells presented an over expression of glial fibrillary acidic protein (GFAP), cell enlargement similarly to astroglial reactivity, functional and structural alterations of cilia and stimulation of SV-NSC proliferation.

There is evidence for ependymal alterations in NMO. Periependymal inflammation has been observed in MRI studies and is included in the international diagnosis criteria for NMO.^1,18,19^ Moreover, a histopathological study of post-mortem tissues showed granulocyte infiltration, microglial reactivity, sub-ependymal gliosis, ependymal cell loss and morphological alteration in NMO patients.^20^ In this study, 56% of NMO patients presented with loss of AQP4 in the ependyma and 38% presented with complement deposition. However, the role of autoantibodies in this alteration has not been evaluated.

In the present study, we hypothesized that, as for astrocytes, purified antibodies from NMO patients can act, *per se*, as cellular modulators and alter ependymal cells’ morphology and functions.

## Materials and methods

### NMO patient and IgG purification

Plasmapheresis were obtained from patients with NMO spectrum disorder selected from the French NMO cohort NOMADMUS and stored at NeuroBioTec (Biological Resource Centre of the *Hospices Civils de Lyon*). Seven patients tested positive for AQP4-IgGs (named NMO_1_-IgG to NMO_7_-IgG for this study) were included. We also used a pool of control plasma collected from healthy blood donors at *Etablissement Français du Sang* (CTRL-IgG). IgGs were purified from plasma and plasmapheresis on Protein-A Sepharose 4 Fast Flow^TM^ beads (P9424 Sigma-Aldrich), eluted with glycine 0.1 M buffer pH 2.8 and then neutralized in Tris 1.5 M pH 8.8. Sample titres ranged from 5 to 10 mg/mL and were diluted to 0.2 mg/mL prior to using in primary cultures and explants of adult rat lateral ventricles. To evaluate their ability to bind to AQP4, NMO-IgGs were incubated for 24 hours on ependymal cells from primary cultures of ependymocytes and on ventricles from adult Sprague Dawley rats’ brain slices and co-stained for human IgG and AQP4 (Supplementary Fig. S1).

### Primary culture of rat ependymocytes

Primary cultures of ependymocytes were obtained as previously described.^21,22^ Up to six brains of freshly born, Sprague Dawley pups were quickly dissected to isolate ventricles and pulled into MEM (1 g/L glucose, no L-glutamine, 21090 ThermoFisher Scientific) supplemented with bovin serum albumin 50 µg/mL (A0281 Sigma-Aldrich), bovin insulin 6.25 µg/mL (I1882 Sigma-Aldrich), human transferrin 12.5 µg/mL (T1283 Sigma-Aldrich), 1% penicillin/streptomycin (15140-122 Gibco) and fungizone 40 µg/mL (Amphotericin B 15290-026 Gibco). Brains were rinsed three times with the same medium and were gently mechanically dissociated by five passages in a G-18 syringe and five passages in a G-21 syringe. Dissociated brains were diluted in 4 mL of medium/brain and seeded on coated culture supports. Dlux 2 chamber culture slices (30402 SPL Life Science) were coated with 40 µg/mL bovin fibronectin (33010-018 Gibco) for 2–3 hours in a 37°C 5% CO_2_ incubator, rinsed one time with MEM and air dried for 45 minutes. The culture medium was first changed 2 days later with MEM supplemented as described previously, with 0.5% penicillin/streptomycin and fungizone 2.5 µg/ml and then changed twice a week. Human thrombin 0.5 IU/ml (T6884 Sigma) was added from days 2 to 14 to increase cell growth.

### Explant culture of adult rat lateral ventricular walls

For the explant culture of dissected rat lateral ventricular wall whole mounts, 2-month-old male Sprague Dawley rats (JanvierLabs) were deeply anesthetized with 5% isoflurane and guillotined. Whole mount dissection of adult rat lateral ventricles was performed as previously described.^23^ Briefly, brains were placed on a petri dish covered with a plastic polymer (Sylgard 184) under a stereomicroscope in 37°C heated L15 Leibovitz medium (114155 Gibco). Hemispheres were separated and a small portion of the posterior part of the brain was removed, allowing the separation of the hippocampus and the neocortex. The hippocampus and the neocortex were gently detached until the extraction of the hippocampus. This manipulation allowed for isolation of the lateral ventricles, which were then isolated. Finally, ventricular wall whole mounts were cultured during 24 hours with or without IgG treatment at 0.2 mg/ml in DMEM Glutamax (61965 Gibco) + 1% penicillin/streptomycin +10% foetal calf serum in 12 well plates in a 37°C and 5% CO_2_ incubator. After 24 hours of treatment, whole mounts were used for an ependymal flow assay or for immunolabelling.

### Ependymal flow assay

Whole mounts treated for 24 hours in explant cultures (see the procedure above) were placed and fixed with needles on a petri dish with Sylgard 184 filled with 37°C L15 Leibovitz under a video macroscope (AxioZoom V16, Zeiss). Borosilicate glass capillaries (1.0 mm O.D.×0.58 mm I.D.) were pulled and processed to obtain a 100 µm internal diameter bevelled tip. Capillaries were placed in an oil microinjector (Narishige) placed on a micromanipulator and filled with fluorescent microbeads (Yellow Green 2 µm diameter latex microspheres, 18338-5, Polysciences). The position of the tip was adjusted on the whole mount using the macroscope. A magnification of 50 × and a resolution of 512 × 512 pixels were used for the recording using the Zen software (Zeiss). The time exposition was adjusted to capture a maximum of image/s (5 fps). At each sequence of video recording, a quantity of about 0.02 µl of the solution containing the microbeads was applied locally, slightly above the surface of the ventricle. After video recording, some whole mounts were fixed for immunolabelling.

### Immunolabelling

The procedure for the immunolabelling of primary ependymocyte cultures and whole mounts of lateral ventricles were very similar and differed only for the duration of certain steps. Cultured cells were fixed with 4% PFA in 0.1 M phosphate buffer for 10 minutes at room temperature and 20 minutes for whole mounts, or with methanol at −20°C for 10 minutes for cells or whole mounts, depending on the antigen sensitivity to the fixation method. Cells were rinsed three times for 5 minutes and whole mounts three times for 15 minutes with 1 × phosphate buffer saline (PBS). Then, samples were incubated with a saturating solution composed of 1 × PBS with or without 0.1–0.3% Triton X-100 (PBST 0.1–0.3%), depending on antigen sensitivity, and 10% normal goat serum (NGS, S-1000, Eurobio). Samples were incubated with primary antibodies diluted in a saturating solution overnight at 4°C. Cells were rinsed three times for 5 minutes with PBST 0.1–0.3%, and whole mounts were rinsed twice quickly and three times for 20 minutes with PBST 0.1–0.3% at room temperature. Samples were then incubated with secondary antibodies diluted in a saturating solution for 1 hour at room temperature. Targets, hosts, references and dilutions of primary and secondary antibodies are listed in Table 1. Cells were rinsed twice with PBST 0.1–0.3% and one time with PBS for 10 minutes and wholemounts were rinsed two times quickly and twice with PBST 0.1–0.3% and once with PBS for 20 minutes at room temperature. Nuclei were stained with DAPI. Finally, samples were mounted with Permafluor (TA-030-FM, ThermoFisher Scientific) and covered with coverslips for microscopy.

After immunolabelling of whole mounts, further steps of dissection were performed to obtain a piece of tissue 200–300 µm thick for slide mounting. Remaining pieces of tissue around the ventricles were cut and the lateral ventricles were gently separated from the rest of the brain by cutting with a short knife just behind them. Whole mounts were then placed on slides, embedded with mounting medium (Permafluor TA-030-FM, ThermoFisher Scientific) and covered with coverslips.

### Isolation of ependymal cells from primary cultures for RNA sequencing

After 24 hours of treatment with CTRL-IgG or NMO-IgG, ependymal cells from primary cultures were rinsed with 1 × Hank’s Balanced Salt Solution (Gibco 14065), detached with accutase (A6964 Sigma) for 5–10 minutes at 37°C, then centrifuged 5 minutes at 1400 rpm and suspended in 1 × PBS (≈10 000 cells/ml). Viable cells were selected by fluorescence activated cell sorting and sorted with propidium iodide (50 µg/ml) at the cytometry platform of the Lyon Cancer Research Centre (CRCL—Inserm U1052—CNRS 5286). The most common marker for mature ependymal cells is FoxJ1.^14^ However, FoxJ1 expression is nuclear rending its isolation by fluorescence activated cell sorting not suitable for RNA sequencing. Since we quantified ∼91% of FoxJ1^+^ cells in our primary cultures (Supplementary Fig. S2), we decided to select viable propidium iodide^+^ cells for RNA sequencing. After cell sorting, cells were kept in the RL buffer (Norgen 51800) at −20°C prior to RNA extraction.

### RNA sequencing and bioinformatic analyses

RNA from primary cultures was isolated and purified using spin column chromatography with the single cell RNA purification kit (Norgen 51800). Library construction was carried out from 5 ng of total RNA using the NEBNext® Single Cell/Low Input RNA Library Prep Kit for Illumina® (NEB) according to the manufacturer’s instructions. Each group of libraries was applied to an Illumina flow cell with high output and run on the Illumina NextSeq500 as a 75 pb single read. Image analysis and base calling were carried out using the NCS 2.0.2 and RTA 2.4.11 Illumina software suites implemented on the Illumina sequencing machine. Final file formatting, demultiplexing and fastq generation were carried out using Bcl2fastq v2.17.1.14. Trimming of reads was performed using Cutadapt software (version 1.9.1—with parameters *-m 50 -q 30,30*).^24^ The abundance of transcripts was estimated by Kallisto software (version 0.44.0—with parameters *–bias*)^25^ using the *Rattus norvegicus* transcriptome (Ensembl release 101—Rnor_6.0—cDNA). Then, differentially expressed transcripts were identified using the R package DESeq2, which implements a negative binomial model and a Wald test on the data normalized by an internal median ratio procedure (version 1.26.0—with default parameters and the following thresholds: log2FoldChange (log2FC) ≥ 1.5 or ≤ 1.5 and adjusted *P* (*P_adj_*) ≤ 0.05).^26^ Functional analysis was performed by Gene Set Enrichment Analysis (GSEA) with the GSEA software (version 4.1.0–1000 permutations, weighted enrichment statistics, gene set exclusion max size of 500 and min size of 15)^27^ with gene sets from the MSigDB (version 7.2—gene set collections: h.all, c2.cp.reactome, c5.go.bp, c5.go.mf and c7.all).^27^ Enrichment map visualization of GSEA was produced with Cytoskape software (version 3.8.2)^28^ and the EnrichmentMap app (Node Cut-off Q-value 0.05 and Edge Cut-off 0.5).^29^

### RNA isolation and quantitative real time PCR

After 24 h treatment, ependymal cells were washed with D-Phosphate Buffered Saline and were collected using TRI-Reagent® RNA/DNA/Protein Isolation Reagent (Molecular Research Centre) treatment. Total cellular RNAs were extracted using the TRI-Reagent® and RNAs were purified by removing genomic DNA using Turbo-DNA-Free Purification Kit (QIAgen). RNA purity and concentration were determined spectrophotometrically using a BioDrop. Nineteen nanograms of total RNA was reverse transcribed to cDNA using the Prime Script RT Reagent Kit (TAKARA OZYME) using Oligo dT primers and random hexamer primers. Quantitative PCR was performed on a Roche Light Cycler 480 using the Light Cycler 480 SYBR Green 1 master mix. PCR amplification of cDNA was carried out by using a synthetic and nonhomologous poly(A) standard RNA (smRNA) in order to scale the efficiency of the reverse-transcriptase.^30^ Standard curves were generated using serial dilutions of primers designed by Eurogentec©. Crossing points on the amplification curves for experimental samples were converted to log copies by comparison with the standard curve. Primers for 12 cytokine and chemokine genes were used (Table 2) and selected based on RNAseq differential analysis.

### Statistical analyses

All analyses of the microscopy images were performed in a blinded fashion. Image analyses were performed with the Fiji software (National Institutes of Health). Graphs and statistical tests were done using Prism 7.0 GraphPad.inc software and R software version 4.2.1 under Rstudio environment.^31^ Linear mixed effects models were built to account for replication with the package ImerTest,^32^. For AQP4 agglomeration and Cx43 particle size, models were built with treatment as a fixed effect, the culture’s well as a random intercept (the number of cultures was too small to consider a random effect) and for Cx43 particles, the number of cells as a fixed effect. For explants where the cell area, the bead speed and directionality, the mean basal patch angle deviation, the number of B cell processes and the number of Ki67 cells were measured, models were built with an explant effect nested within a rat effect as random intercepts and treatment as a fixed effect. The Gaussian distribution of residuals and random effects was assessed visually with a quantile-quantile plot for all models. For statistical analysis of angle distribution, the CircStats package was used.^33^ For the cytokine analysis, a non-parametric Kruskal–Walli’s test was used considering the non-normal distribution (Shapiro–Wilk test) of the data. Dunnet’s post-hoc multiple comparison tests were performed to reveal differences between non-treated (NT) groups and IgG treated groups using Prism 7.0 GraphPad.inc software. Graphs are plotted as medians + 95% confidence interval (CI) histograms superposed to the mean per culture or explant dot plots. *P*-values < 0.05 were considered statistically significant and the level of significance is indicated as follows: * = *P* ≤ 0.05; ** = *P* ≤ 0.01 and *** = *P* ≤ 0.001.

## Results

### Purified IgG from NMO patients induces AQP4 expression alteration and cell enlargement in ependymal cells

**Figure 1:**
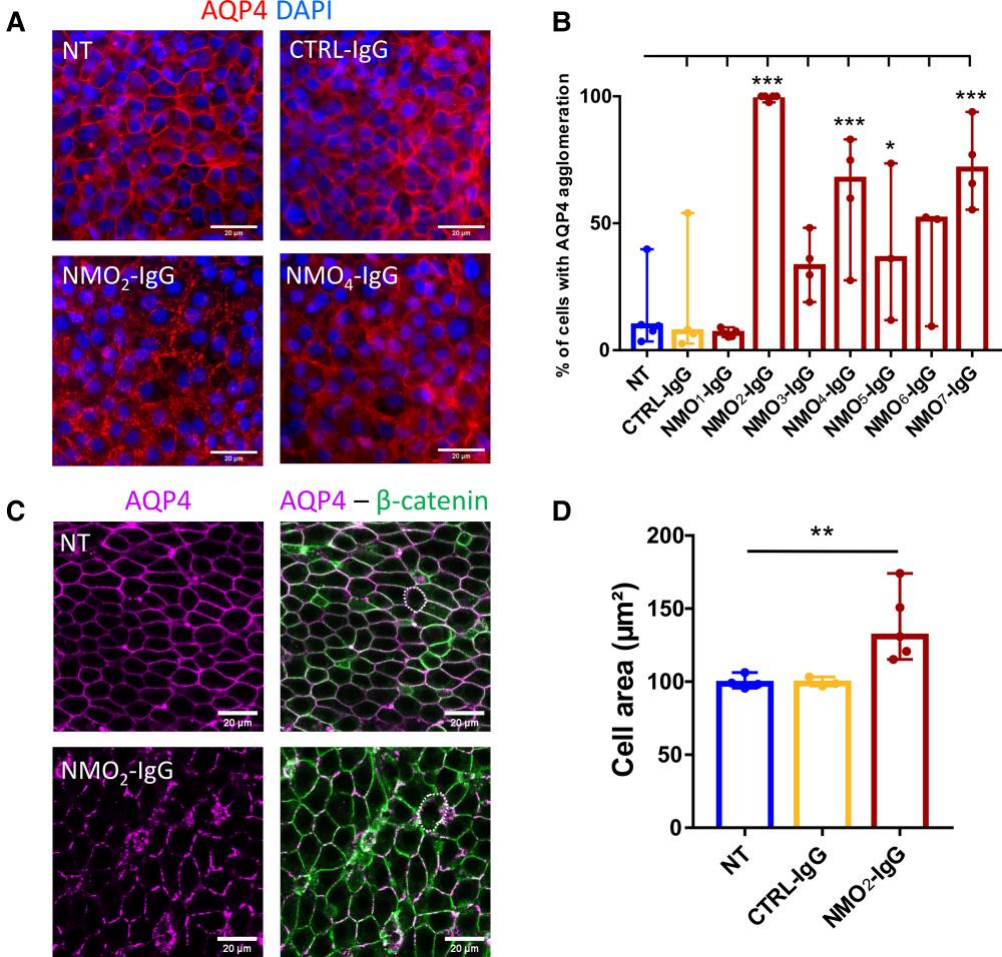
**NMO-IgG-induced AQP4 agglomeration and ependymal cell enlargement.** (**A**) Microphotographies of primary ependymal cell cultures after 24 hours treatment with CTRL-IgG, NMO_2_-IgG, NMO_4_-IgG or NT cells labelled for AQP4 (red) and DAPI (blue). In NT and CTRL-IgG conditions, AQP4 is uniformly expressed at the lateral membranes, but not in NMO_2_-IgG, where AQP4 is agglomerated. NMO_4_-IgG presented an intermediate effect on AQP4 agglomeration. (**B**) The percentage of cells presenting AQP4 agglomeration in microphotographies was increased for 4/7 NMO-IgG (2, 4, 5 and 7) compared with NT (histogram = median + 95% CI, *n* = 2–5 cultures × 10 fields analysed/condition). Mixed effects model with treatment as fixed effect compared with NT: CTRL-IgG: *P* = 0.64, NMO_1_-IgG: *P* = 0.49, NMO_2_-IgG: *P* = 5.09e-09, NMO_3_-IgG: *P* = 0.14, NMO_4_-IgG: *P* = 3.16e-04, NMO_5_-IgG: *P* = 0.048, NMO_6_-IgG: *P* = 0.077, NMO_7_-IgG: *P* = 1.23e-05. (**C**) Confocal images of whole mount lateral ventricle wall explants cultured and treated during 24 hours with CTRL-IgG or NMO_2_-IgG labelled for AQP4 (magenta) and β-catenin for lateral membranes (green). (**D**) Histogram of the median ± 95% CI of the cell area (µm²) of ependymal cells. Cell area was measured [dot circles as an example in (**C**)] in confocal images and was increased by NMO_2_-IgG (NT: *n* = 4, CTRL-IgG: *n* = 3, NMO_2_-IgG: *n* = 5 explants × 5 fields analysed/explant × 30 cells analysed/field). Mixed effects model with treatment as a fixed effect compared with NT: CTRL-IgG: *P* > 0.99 and NMO_2_-IgG: *P* = 4.12e-03.

To evaluate the effects of NMO-IgG on AQP4 expression in ependymal cells, primary cultures were treated for 24 hours with NMO-IgG purified from seven different NMO patients (called NMO_1_-IgG to NMO_7_-IgG) and compared with NT or CTRL-IgG (a purified pool from healthy donors) conditions. In NT and CTRL-IgG conditions, AQP4 was distributed uniformly along lateral membranes (Fig. 1A). After NMO-IgG treatment, AQP4 was aggregated at the lateral membrane for four of seven patients with the NMO_2_-IgG with the strongest effect (Fig. 1A and B). Interestingly, this patient was also the only one presenting periependymal lesion around the lateral ventricles and fourth ventricle visible on cerebral MRI performed before plasma exchange (Supplementary Fig. S3). Treatments of different durations with NMO_2_-IgG were tested (1, 3, 24 and 48 hours). AQP4 started to agglomerate at 3 hours for NMO_2_-IgG, but a clearer result was visualized at 24 hours (Supplementary Fig. S4). Cultured ependymal cells appeared to increase in size after NMO-IgG treatment. To confirm this increase in size, we examined dissected whole mounts of adult rat lateral ventricles that were exposed for 24 hours to NMO_2_-IgG and fixed for immunolabelling and visualization by confocal microscopy. We only used NMO_2_-IgG to reduce the number of animals used. In NT or CTRL-IgG conditions, AQP4 was also expressed at the lateral membrane of ependymal cells and as expected, NMO_2_-IgG-induced AQP4 agglomeration (Fig. 1C). A clear difference in cell size was found in the NMO_2_-IgG condition, compared with the control conditions (Fig. 1D, NT = 101.44 ± 2.34; CTRL-IgG = 99.67 ± 2.23; NMO_2_-IgG = 139.83 ± 3.54 µm²). To summarize, *in vitro* and *ex vivo* experiments showed that NMO-IgG-induced AQP4 agglomeration in a patient-dependent manner and cell size enlargement.

### Purified IgG from NMO patients induce alteration of gap junction expression in ependymal cells

**Figure 2:**
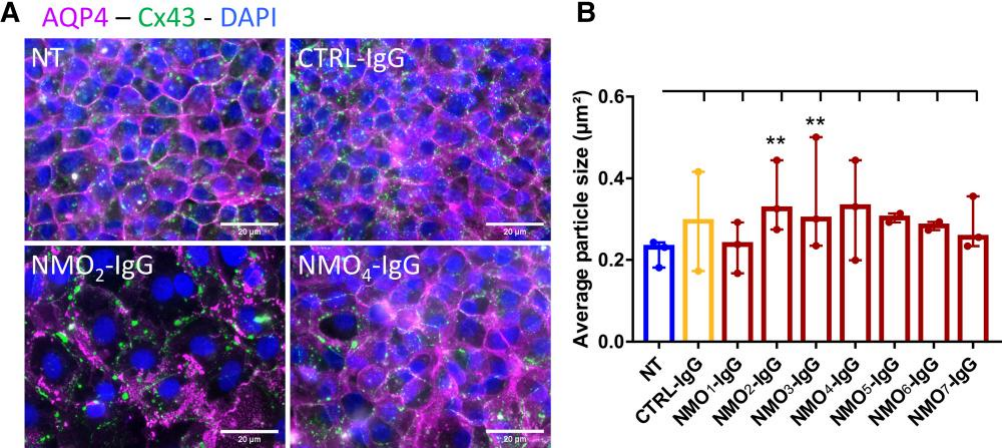
**NMO-IgG altered gap junction expression in primary cultures.** (**A**) Microscope images of ependymal cells labelled for AQP4 (magenta) and Cx43 (green) after 24 hours of treatment with CTRL-IgG or NMO-IgG showing Cx43 particle’s increased size. (**B**) Histogram of the median ± 95% CI of the mean Cx43 particle size (µm²) after 24 hours of treatment. NMO-IgG increased Cx43 particle size in a patient-dependent manner (*n* = 2–3 cultures × 10 fields analysed/condition). Mixed effects model with treatment as fixed effect compared with NT: CTRL-IgG: *P* = 0.086, NMO_1_-IgG: *P* = 0.78, NMO_2_-IgG: *P* = 0.025, NMO_3_-IgG: *P* = 0.027, NMO_4_-IgG: *P* = 0.058, NMO_5_-IgG: *P* = 0.49, NMO_6_-IgG: *P* = 0.72, NMO_7_-IgG: *P* = 0.24.

Gap junctions are important molecular supports of ependymal cell functions, and gap junction alterations have been linked to astrocyte dysfunction induced by NMO-IgG.^12^ To test if gap junctions are also affected by NMO-IgG in cultured ependymal cells, we analysed the effects of several NMO-IgG on the expression of Connexin 43 (Cx43), the main gap junction of ependymal cells, on primary cultures after 24 hours treatment.^34^ In NT cultures, Cx43 was expressed as agglomerates at the lateral membrane (Fig. 2A). Compared with NT or CTRL-IgG treated cells, NMO-IgG increased Cx43 spot size in a patient-dependent manner (Fig. 2B, NT = 0.218 ± 0.035; CTRL-IgG = 0.295 ± 0.069; NMO_1_-IgG = 0.233 ± 0.029; NMO_2_-IgG = 0.348 ± 0.042; NMO_3_-IgG = 0.346 ± 0.067; NMO_4_-IgG = 0.325 ± 0.053; NMO_5_-IgG = 0.303 ± 0.039; NMO_6_-IgG = 0.283 ± 0.031; NMO_7_-IgG = 0.282 ± 0.038 µm²). These results suggest that NMO-IgG modulates Cx43 expression, reducing gap junction communication.

### Purified IgG from NMO patients induce ependymal cell cilia motility impairments and planar polarity disruption

**Figure 3:**
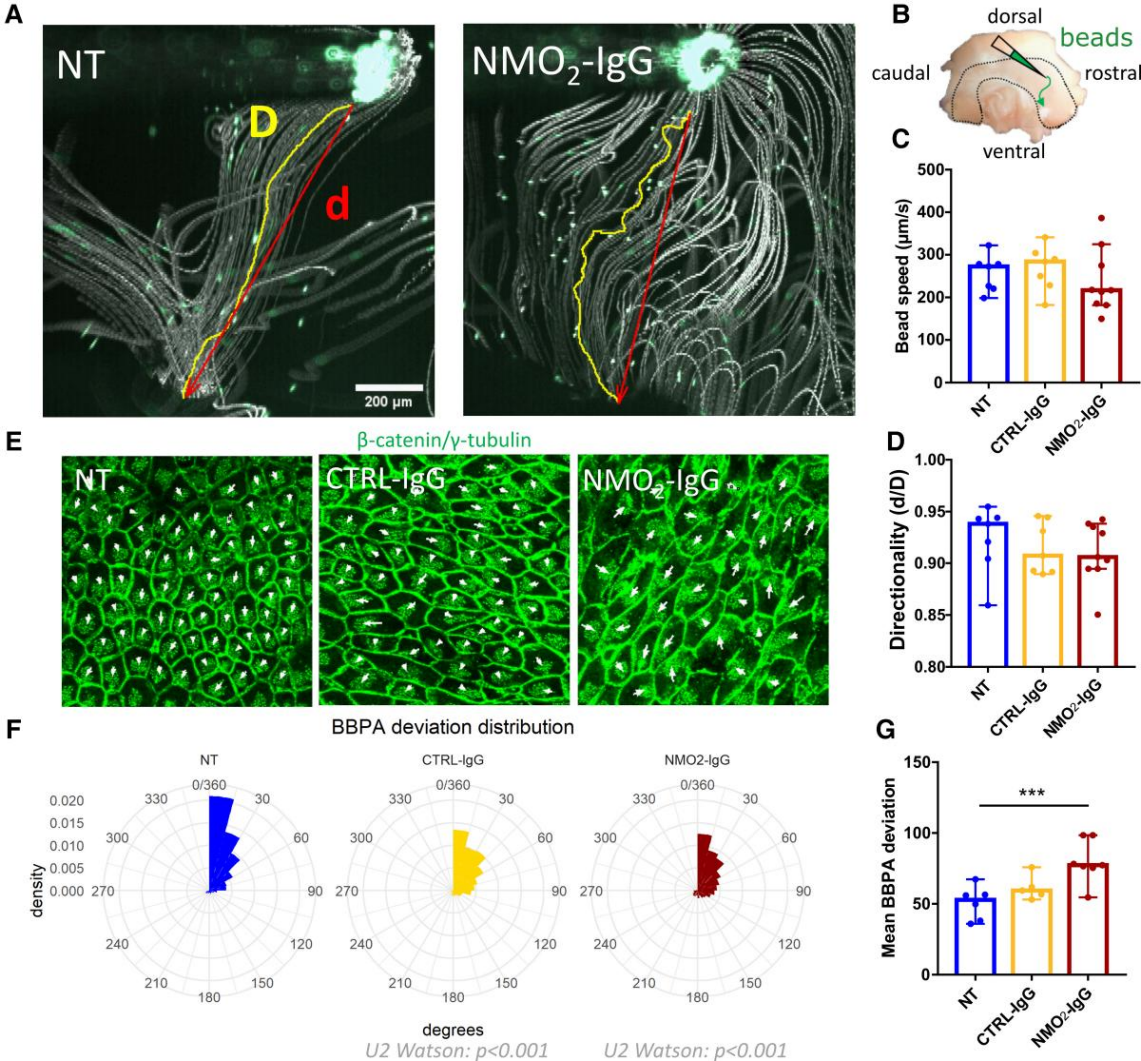
**NMO-IgG alters cilia motility and planar polarization in lateral wall explant culture.** (**A**) Video macroscopic overtime stacks of fluorescent microbead trajectories applied on the surface of cultured whole mounts after 24 hours of treatment. The trajectory (D, yellow line) and the distance between the start and end points of cilia propelled beads (d, red line) were measured. (**B**) Schematic view of the bead deposition localization on the whole mounts. Histogram of the median ± 95% CI showing that the bead's speed (µm/s) (**C**) and the directionality (d/D) (**D**) were reduced after NMO_2_-IgG treatment compared with NT and CTRL-IgG (NT: *n* = 7 explants, CTRL-IgG: *n* = 8 explants and NMO_2_-IgG: *n* = 9 explants × 30 beads analysed/condition). (**C**) Mixed effects model with treatment as a fixed effect compared with NT: CTRL-IgG: *P* = 0.89 and NMO_2_-IgG: *P* = 0.49. (**D**) Mixed effects model with treatment as a fixed effect compared with NT: CTRL-IgG: *P* = 0.54 and NMO_2_-IgG: *P* = 0.38. (**E**) Confocal images of cultured lateral wall explants treated for 24 hours with CTRL-IgG or NMO_2_-IgG. Cilia basal bodies were labelled with γ-tubulin and intercellular junctions with β-catenin (both green). Vectors were drawn between cell centre and basal body patches (arrows). (**F**) BBPA deviations to the median of each field are plotted on radial charts (angles in degrees). The distribution of BBPA in NT was uniform (Watson’s test for circular uniformity: *U* = 46.7227; *P* < 0.001) and was heterogeneously distributed in CTRL-IgG (*n* = 5 explants) and NMO_2_-IgG (*n* = 7 explants) conditions compared with NT (*n* = 6 explants, Watson's two-sample test of homogeneity: NT versus Ctrl-IgG: *U2* = 0,6417; *P* < 0001; NT versus NMO-IgG: *U2* = 2,5419; *P* < 0,001, 5 fields analysed/condition). (**G**) Histogram of the median ± 95% CI showing that the mean BBPA deviation from median was significantly different between the NMO_2_-IgG and NT (NT: *n* = 6 explants, CTRL-IgG: *n* = 5 explants and NMO_2_-IgG: *n* = 7 explants × 5 field averaged BBPA). Mixed effects model with treatment as a fixed effect compared with NT: CTRL-IgG: *P* = 0.14 and NMO_2_-IgG: *P* = 8.43e-04.

Cilia are of utmost importance for the regulation of ependymal cell functions. To test if NMO-IgG functionally alters the motility of ependymal cell cilia, we performed an ependymal flow assay by depositing a small amount of fluorescent microbeads on the surface of whole mounts of adult rat lateral walls, after 24 hours of treatment with NMO_2_-IgG or CTRL-IgG. We recorded the bead’s trajectories using a video macroscope (Videos 1 and 2) and measured the speed and directionality of the bead diffusion. Directionality was defined as the ratio between the distance between the first position and the last position of the bead (d) on the total length of bead’s trajectory (D), and the speed was calculated by dividing D by the time (Fig. 3A). In NT conditions, beads deposited as shown in the scheme (Fig. 3B) were propelled ventro-rostrally (Fig. 3A). In NT and CTRL-IgG conditions, beads’ speeds were 256.8 ± 39.7 and 268.7 ± 48.7 µm/s, respectively, whereas NMO_2_-IgG reduced the bead’s speed to 239 ± 58.3 µm/s compared with both NT and CTRL-IgG (Fig. 3C). Similarly, NMO_2_-IgG also reduced the directionality in comparison to NT (Fig. 3D, NT = 0.924 ± 0.03; CTRL-IgG = 0.915 ± 0.02; NMO_2_-IgG = 0.910 ± 0.02).

Ependymal planar polarity, which is the orientation of motile cilia from neighbouring cells in the same direction, is essential for the proper propelling of CSF or microbeads.^35,36^ To test if NMO-IgG alters the planar organization of ependymal cilia, we examined the planar polarity on the ventricular wall whole mounts after 24 hours of treatment with CTRL-IgG and NMO_2_-IgG labelled for β-catenin (a marker of intercellular junctions) and γ-tubulin (a marker of ciliary basal bodies) using confocal microscopy. Basal body patch angles (BBPA) were measured by drawing arrows from the centre of ependymal cells towards basal body patch centres. For each confocal image, each BBPA was subtracted to the median of the field. The planar polarity was measured as the distribution of BBPA deviations from median. In NT whole mounts, the distribution of BBPA deviations from median was not uniform, confirming polarity (Fig. 3E and F). For CTRL-IgG and NMO_2_-IgG treated whole mounts, the distribution of BBPA deviations to median was dispersed in comparison to NT whole mounts meaning polarity disruption (Fig. 3E and F). The mean BBPA deviation was significantly different between NT and NMO_2_-IgG but not CTRL-IgG treated whole mounts (NT = 50.39 ± 8.61°; CTRL-IgG = 61.39 ± 7.85°; NMO_2_-IgG = 77.18 ± 8.04°) suggesting that the planar polarity disruption is stronger for NMO_2_-IgG than for CTRL-IgG (Fig. 3E). Globally, we showed that NMO-IgG altered cilia motility and the planar polarization of cilia tufts but did not alter the structural morphology of cilia (data not shown). In conclusion, NMO-IgG may impair the cilia’s ability to propel CSF by disrupting the orientation of cilia tufts.

### Activation of sub-ventricular neural stem cells proliferation after exposure to purified IgG from NMO patients

**Figure 4:**
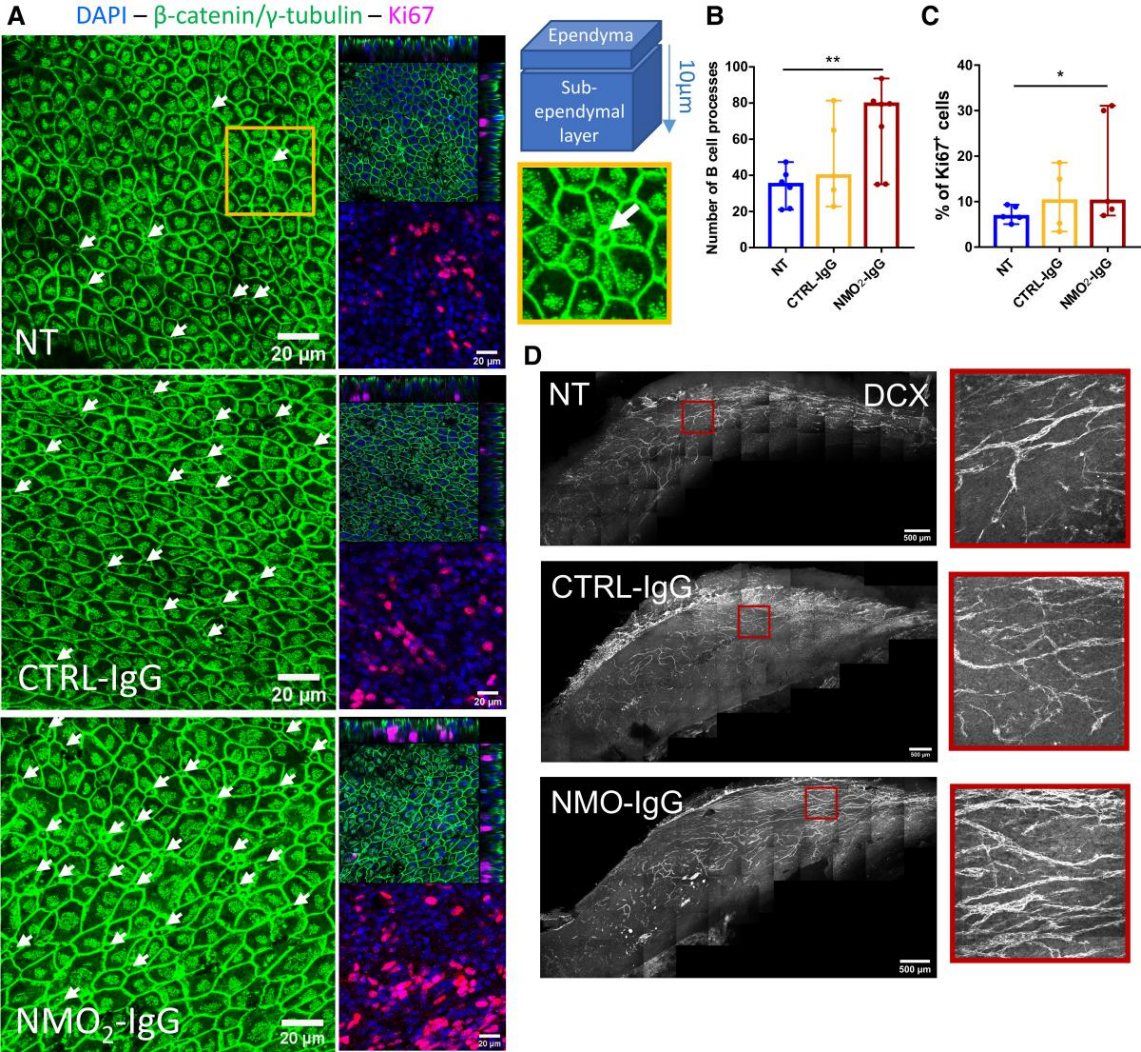
**NMO-IgG induced sub-ventricular stem cell proliferation in lateral wall explant culture.** (**A**) Confocal images of ventricular wall whole mounts treated for 24 hours with CTRL-IgG or NMO_2_-IgG and labelled for β-catenin and γ-tubulin (green), Ki67 (magenta) and DAPI (blue). B1 qNCS processes were identified with their monocilium (arrows) in the centre of pinwheels (yellow square). Sub-ependymal Ki67^+^ was counted until 10 µm under the ependymal layer (as shown in the orthogonal views and in the schematic). (**B**) Histogram of the median ± 95% CI showing that NMO_2_-IgG increased the number of B1 NSCs processes compared with NT and CTRL-IgG (NT: *n* = 6, CTRL-IgG: *n* = 5 and NMO_2_-IgG: *n* = 7 explants × 5 fields analysed/condition). Mixed effects model with treatment as a fixed effect compared with NT: CTRL-IgG: *P* = 0.089 and NMO_2_-IgG: *P* = 5.77e-03. (**C**) Histogram of the median ± 95% CI showing that the number of proliferating sub-ventricular cells was significantly increased by NMO_2_-IgG compared with NT (NT: *n* = 5, CTRL-IgG: *n* = 4, NMO_2_-IgG: *n* = 5 explants × 5 fields analysed/condition). Mixed effects model with treatment as fixed effect compared with NT: CTRL-IgG: *P* = 0.14 and NMO_2_-IgG: *P* = 0.012. (**D**) Tiled confocal images of cultured lateral wall explants after treatment with CTRL-IgG and NMO_2_-IgG labelled for double cortin allowing visualization of the migratory neuroblast network. The double cortin network was denser after NMO_2_-IgG treatment, as shown in the zoomed images in the red squares.

The SV-NSCs have been shown to proliferate in different neuroinflammatory conditions.^16,17^ However, the response of SV-NSCs is still unclear in NMO models.^37^ Here, we evaluated the effects of NMO-IgG treatment during 24 hours on whole mount explant cultures. In the ependymal layer, quiescent neural stem cells (qNSCs) called B1 cells are able to produce precursors for neuroblasts and glial cells and project one process harbouring a single sensory cilium that is easily identified at the centre of ependymal surrounding pinwheels (Fig. 4A, yellow square).^34^ After 24 hours of treatment, NMO_2_-IgG increased the number of B1 processes compared with NT and CTRL-IgG conditions (Fig. 4A, B; NT = 33.79 ± 5.88; CTRL-IgG = 48.83 ± 10.65; NMO_2_-IgG = 68.21 ± 8.39 number of B1 processes). The number of proliferating cells under the ependymal layer was measured with the marker of proliferation Ki67. The percentage of sub-ventricular Ki67 cells was increased after NMO_2_-IgG treatment compared with NT and CTRL-IgG treatment (Fig. 4A–C, NT = 7.29 ± 1.48%; CTRL-IgG = 10.55 ± 3.55%; NMO_2_-IgG = 17.49 ± 5.45%). Thus, NMO-IgG may activate qNSCs or other progenitor cells to produce neural progenitors in the sub-ventricular zone. Most cells produced by the SVZ in rodents are neuroblasts, and their migration is dependent on ciliary beating and polarity.^38^ Therefore, cultured explants were labelled for doublecortin, allowing visualization of the migratory neuroblast network. After treatment with NMO_2_-IgG, the network was denser compared with NT or CTRL-IgG conditions (Fig. 4D). These results suggest that NMO-IgG increased the proliferation of migrating neuroblasts in the sub-ventricular zone.

### Inflammatory response induced by purified IgG from NMO patients in ependymal cells

We performed a transcriptional analysis by RNA sequencing of ependymal cells from primary cultures and compared NT, CTRL-IgG and NMO_2_-IgG treated cells. CTRL-IgG significantly increased the expression of 118 transcripts and decreased the expression of 80 transcripts compared with NT, and NMO_2_-IgG increased the expression of 295 transcripts and decreased the expression of 70 transcripts compared with NT (Supplementary Fig. S5A and B). In all the genes differentially expressed by CTRL-IgG and NMO_2_-IgG, 27% were common, 18.5% were specific to CTRL-IgG and 54.5% were specific to NMO_2_-IgG (Supplementary Fig. S5C). Even if a high proportion of transcripts were commonly up-regulated or down-regulated, these results suggest that ‘healthy’ IgGs induced a specific transcriptomic response by ependymal cells that was not found for the NMO pathogenic ones. To investigate the functional differences between CTRL-IgG and NMO_2_-IgG compared with NT, we performed a GSEA. NMO_2_-IgG up-regulated gene sets related to immune system activity (23 gene sets more than CTRL-IgG), cell cycle regulation, cellular response to stress and gap junction assembly (Supplementary Fig. S5D). Interestingly, we found one gene set related to intraciliary transport down-regulated in NMO_2_-IgG versus NT (Supplementary Fig. S6A, *FDR q-value* = 0.63 and *P* = 0.002, online resource), with a significant decrease in the log2FC for Lca5 (Supplementary Fig. S6B, NT versus NMO_2_-IgG (*P_adj_* < 0.01, online resource), NT versus CTRL-IgG (non-significant) and NMO_2_-IgG versus CTRL-IgG (*P_adj_* < 0.001)).

**Figure 5:**
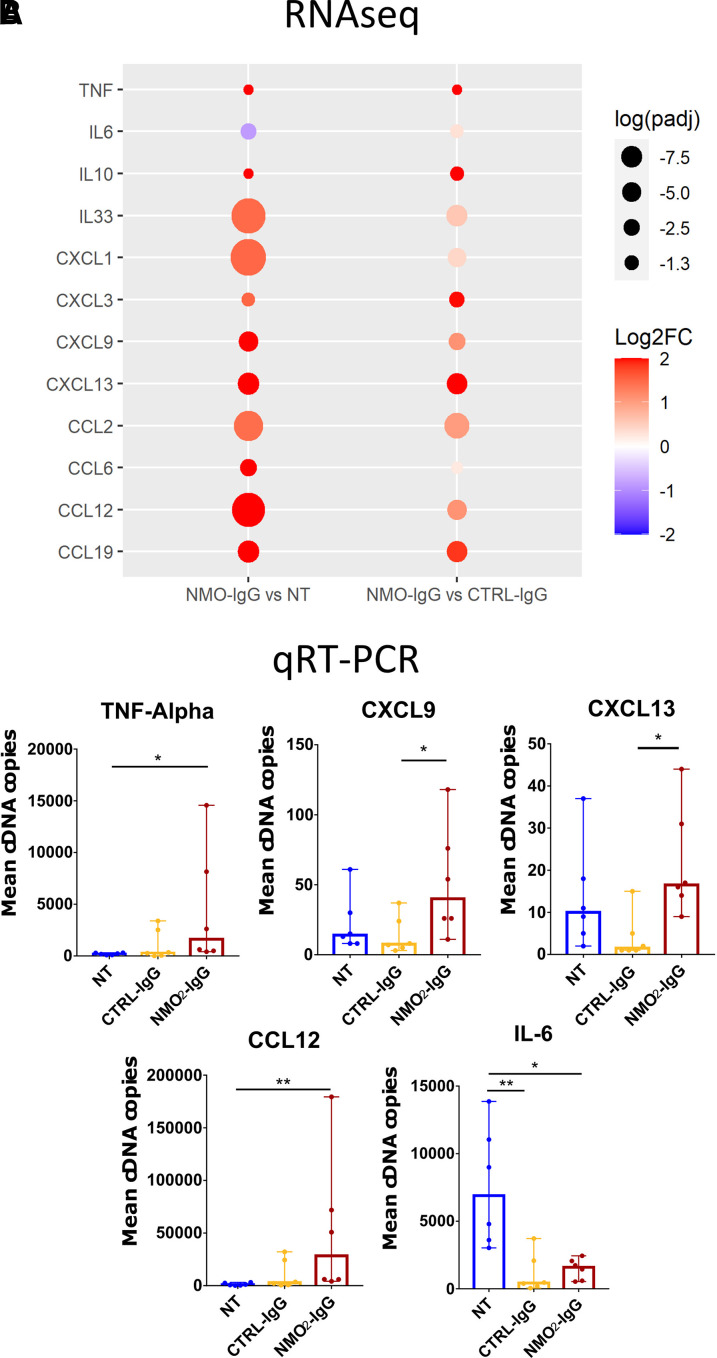
**NMO_2_-IgG induced the expression of a specific panel of cytokines in primary cultures.** (**A**) Dot-plot showing the different cytokines up-regulated or down-regulated in at least one comparison between NMO_2_-IgG versus NT, CTRL-IgG versus NT or NMO_2_-IgG versus CTRL-IgG treated primary ependymal cell cultures (|log2FC| ≥ 1.5 or *P_adj_* ≤ 0.05 (log (0.05) = 1.3)). NMO_2_-IgG increased TNF-α, IL10, IL33, CXCL1, CXCL3, CXCL9, CXCL13, CCL2, CCL6 and CCL19 compared with NT and CTRL-IgG, and down-regulated IL6 compared with NT but not CTRL-IgG. (**B**) Histogram of the median ± 95% CI of the number of cDNA copies obtained by qRT-PCR after 24 hours treatment with CTRL-IgG or NMO_2_-IgG for the cytokines selected in A. IL6 was significantly down-regulated by CTRL-IgG and NMO_2_-IgG (**P = 0.006, *P = 0.04) and TNF-α (*P = 0.02), CXCL9 (*P = 0.04), CXCL13 (*P = 0.02) and CCL12 (**P = 0.006) were significantly up-regulated by NMO_2_-IgG (*n* = 3 cultures, 2 duplicates/culture, Kruskal–Wallis and Dunn’s multiple comparison tests)

Since there was a strong up-regulation of gene sets related to inflammatory processes revealed by the GSEA, we then determined whether NMO-IgG induces a chemokine/cytokine response of ependymal cells by analysing the RNAseq data and using quantitative real time PCR (qRT-PCR). In the RNAseq differential analysis, a panel of 11 cytokines has been selected (Fig. 5A; |log2FC| ≥ 1.5 or *P_adj_* < 0.05 in at least one comparison (NMO_2_-IgG versus NT, CTRL-IgG versus NT or NMO_2_-IgG versus CTRL-IgG). NMO_2_-IgG increased TNF, IL10, IL33, CXCL1, CXCL3, CXCL9, CXCL13, CCL2, CCL6 and CCL19 and down-regulated IL6 compared with NT (Fig. 5A). NMO_2_-IgG up-regulated all these cytokines when compared with CTRL-IgG (Fig. 5A). To confirm these results, we performed qRT-PCR after 24 hours of treatment on primary ependymal cell cultures. IL6 was significantly down-regulated by CTRL-IgG, NMO_2_-IgG, and TNF-α, CXCL9, CXCL13 and CCL12 were significantly up-regulated by NMO_2_-IgG (Fig. 5B).

## Discussion

We showed that purified IgG from NMO patients altered ependymal cell morphology and functions, both on the primary cultures of ependymal cells and on adult rat lateral ventricle explants. This changes included the following: (i) AQP4 agglomeration at the lateral membrane accompanied by an increase of ependymal cell size; (ii) alteration of gap junction expression, with the increase of Cx43 particle size; (iii) depolarization of ciliary basal bodies and alteration of cilia motility in an ependymal flow assay; (iv) activation of the sub-ventricular neural stem cell niche with an increase of B1 processes, an increase of Ki67 dividing cells and densification of the migrating neuroblast network in the sub-ependymal zone; and (v) strong activation of transcriptomic pathways linked to cell proliferation and to immune system activity, associated to a specific cytokine/chemokine profile. In summary, we showed that NMO-IgG triggered a reactive phenotype in ependymal cells as shown by cell enlargement and function impairments, an activation of the sub-ventricular zone and a production of inflammatory cytokines and chemokines.

NMO-IgG binding to AQP4 on astrocytes leads to various outcomes such as cell death and necrosis, down-regulation of AQP4 and GFAP expression, gliosis with GFAP overexpression, or astrocytic dysfunction with preserved AQP4 and GFAP.^6^ These outcomes are highly dependent not only on the disease course and the lesion site in the central nervous system but also on the AQP4 isoform prevailing (M1 or M23).^7,39^ The M23 isoform is the major constituent of the transmembrane structures called orthogonal arrays of particles, visible by electronic microscopy.^40^ NMO-IgG internalizes more easily the M1 isoform than the M23 isoform, whereas the M23 targeting induces the enlargement of orthogonal arrays of particles.^39^ For ependyma, different outcomes were also observed in an animal model and in patient tissue, where ependymal denudation and ependymal preservation with AQP4 loss and complement deposition were co-observed in the same samples.^20,41^ Here, we showed AQP4 agglomeration at the lateral membrane in ependymal cells after NMO-IgG treatment. The expression ratio of the different isoforms of AQP4 in ependymal cells has not been evaluated, but the strong presence of orthogonal arrays of particles on ependymal cells suggests that AQP4 agglomerates might resist internalization in ependymal cells as it does in astrocytes.^42^ The increase in ependymal cell size suggests an impairment of cell volume homeostasis due to an alteration of water regulation. However, such an ependymal cell size was also observed in response to inflammatory stimuli in mice models of experimental autoimmune encephalomyelitis and stroke.^16,17^ Therefore, we cannot assure that the water balance is impaired, and it might rather correspond to a reactive phenotype harboured by ependymal cells.^16,17^ In contrast to the ependymal denudation observed in post-mortem tissue from NMO patients, we did not observe any loss of ependymal cells.^20^ The cell viability was not decreased after NMO-IgG treatment (data not shown) and we did not find activation of apoptotic or necrotic pathways in our RNAseq analysis. The difference may be explained by the fact that we did not use complement in our models and this suggests that although NMO-IgG alone can modulate ependymal cell functions and disorganize AQP4 expression, the complement is necessary to trigger ependymal cell death and denudation.^43^

We found that NMO-IgG impairs cilia motility and basal body planar polarity. Several hypotheses can be proposed for such ependymal dysfunction. First, the agglomeration of AQP4 and the enlargement of ependymal cells could induce cytoskeletal disorganization or provide a non-molecular mechanical signal that induces the delocalization of planar cell polarity regulators.^36,44^ Second, it has been shown that Cx43 expression and calcium waves propagated through gap junctions regulate the maintenance of cilia motility.^15^ Consequently, the gap junction communication deficit we observed in our model could explain by itself the effects on cilia motility. Third, cilia motility deficits and impairment of cilia orientation were observed in other neuroinflammatory models, suggesting a direct impact of inflammation on cilia motility and basal body polarization.^16,17^ Therefore, gliotic activation and inflammation induced in ependymal cells by NMO-IgG could alter cilia motility and basal body orientation. Another hypothesis emerged from the RNAseq data analysis, showing a strong down-regulation of LCA5. LCA5 is involved in Leber congenital amaurosis, which causes childhood blindness via cell death of the retinal photoreceptor.^45^ Knock out of *LCA5* caused photoreceptor dystrophy due to intraciliary transport trafficking deficit. This deficit was linked to the mislocalization of Ift88, a protein regulating intraflagellar trafficking of proteins.^46^ Intraflagellar transport is essential for the development and maintenance of flagella and cilia via the trafficking of cilia components, signalling molecules or membrane channels.^47^ Our results suggest that cytoskeletal or homeostatic changes could impair intraciliary trafficking and thus be linked to cilia motility alteration.

Evaluating the integrity of neurogenic niches in demyelinating diseases is primordial if one wants to stimulate neurogenesis to repair injured areas. Periependymal lesions in NMO suggest that the sub-ventricular niche may be compromised, lacking the ability to produce new cells for tissue repair.^48^ Sera from NMO patients can enhance the production by SVZ neurospheres of GFAP^+^ cells but reduce that of Tuj1^+^ (neurons) and Olig2^+^ (oligodendrocyte precursor cell).^37^ Similarly, we found that NMO-IgG activates the proliferation of sub-ventricular cells and of qNSCs. NMO-IgG might have an effect not only directly on NSCs but also indirectly on targeting ependymocytes and astrocytes, leading to the release of inflammatory cytokines or transcription factors. The increased proliferation of B1 astrocytes could be due to direct targeting by NMO-IgG but not to anti-AQP4 antibodies since these cells do not express AQP4.^49^ Belenguer *et al*. showed that TNF-α regulates VZ-NSCs differentiation and proliferation.^50^ Therefore, the production of TNF-α by ependymal cells in response to NMO-IgG observed in our model could explain the proliferation of qNSCs. In addition, it is known that ciliary beating and the correct propulsion of CSF regulate the organization of the rostral migratory stream of neuroblast produced in the SVZ that migrates towards the olfactory bulb.^38^ Therefore, the deficit in ciliary beating shown in this study suggests that the rostral migratory stream might be impaired, thereby compromising neuroblast migration. Since the migrating neuroblast network was denser, it suggests that the neuroblast might stagnate. Parallelly, reducing CSF circulation may lead to accumulation of transcription factors and inflammatory molecules activating the sub-ventricular niche. However, this overactivation of the niche may lead to long-term exhaustion of precursor cells and reduce neural and glial repair.

Our findings support an early role for ependymocyte impairments in NMO pathogenesis. In agreement, Guo *et al.* ^20^ proposed a suite of events beginning with NMO-IgG altering the choroid plexus’s epithelial cells, expressing AQP4 and favouring the CSF entry of NMO-IgG. Since ependymal cells harbour low levels of tight junctions, NMO-IgG present in the CSF could then easily reach and bind AQP4 expressed at the basolateral membrane of ependymal cells and sub-ventricular astrocytes.^51^ Astrocytes might thus be first targeted and fragilized by NMO-IgG penetrating parenchyma through the ependymal interface, leading to a blood–brain barrier (BBB) weakening from the inside. Our previous work supports this hypothesis. Indeed, we showed accumulation of rat IgG in periventricular areas in an NMO rat model and that purified IgG from NMO patients could alter BBB permeability, suggesting that the entry of rat IgG into parenchyma was due to BBB weakening. Since NMO-IgG was administered directly in the ventricles, BBB weakening could occur internally via the CSF to the parenchyma upon entry of NMO-IgG.^52^ Furthermore, we found an up-regulation of chemokines, namely CXCL9, CXCL13 and CCL12, that are known to stimulate the activation and recruitment of T cells, B cells and macrophages, respectively, and has been shown to be elevated in the CSF of NMO patients or after NMO-IgG treatment of astrocytes.^9,53,54^ Thus, ependymal cells produced pro-inflammatory chemokines that may participate in the recruitment of invading immune cells towards periventricular lesions in the acute phase of NMO. The idea that the CSF presence of NMO-IgG is pathogenic is reinforced by the fact that the detection of NMO-IgG in the CSF during attacks correlates with astrocytic damage, BBB breakdown and the release of pro-inflammatory cytokines and chemokines in patients.^55^ In addition, troubles in CSF circulation could contribute to the NMO pathogenesis by a lack of clearance of toxic wastes and autoantibodies. Furthermore, in an MRI study, the lateral and third ventricle volumes were increased in NMO patients compared with a control group.^56^

This study presented some limits. First, it is probable that in NMO several autoantibodies are involved in the pathophysiology.^57^ In addition, AQP4-Ab autoreactivity is probably polyclonal in human disease. Thus, we decided, on purpose, to use IgG from NMO patients that reflect the diversity of the process and we cannot exclude that some alterations could be mediated by other antibodies in our models. Second, after NMO-IgG treatment *in vitro*, there was an up-regulation of gene sets linked to cell proliferation (Supplementary Fig. S5D). However, brain ependymal cells do not proliferate in physiological conditions and neither in pathological conditions.^58,59^ Since we did not observe proliferation in the *ex vivo* model, this effect might be due to the culture conditions.

## Conclusion

Using original *in vitro* and *ex vivo* models, merging proteomic, transcriptomic and functional evaluation, we showed that ependymal cells in contact with autoantibodies from NMO patients become reactive and display rapid morphological and functional changes that could enhance and facilitate tissue injury. This study provides a new perspective on NMO that should be considered not only as an astrocypathy but also as an ependymocytopathy and opens the path for targeted modulation therapy on ependyma.

## Supplementary Material

fcac307_Supplementary_Data

## Data Availability

The data that support the findings of this study are available from the corresponding author on request.

## Abbreviations

AQP4 =: aquaporin-4
BBB =: blood–brain barrier
CCL =: C-C motif chemokine ligand
CXCL =: C-X-C motif chemokine ligand
CXCR =: C-X-C motif chemokine receptor
GFAP =: glial fibrillary acidic protein
IgG =: immunoglobulin G
IL =: interleukin
NMO =: neuromyelitis optica
qRT-PCR =: quantitative real time PCR
SV-NSCs =: sub-ventricular neural stem cells
TNF =: tumour necrosis factor

## References

[1] Wingerchuk DM, Banwell B, Bennett JL, et al International consensus diagnostic criteria for neuromyelitis optica spectrum disorders. Neurology. 2015;85:177–189. 26092914 10.1212/WNL.0000000000001729 PMC4515040

[2] Lennon VA, Wingerchuk DM, Kryzer TJ, et al A serum autoantibody marker of neuromyelitis optica: Distinction from multiple sclerosis. Lancet. 2004;364:2106–2112. 15589308 10.1016/S0140-6736(04)17551-X

[3] Nielsen S, Nagelhus EA, Amiry-Moghaddam M, Bourque C, Agre P, Ottersen OP. Specialized membrane domains for water transport in glial cells: High-resolution immunogold cytochemistry of aquaporin-4 in rat brain. J Neurosci. 1997;17:171–180. 8987746 10.1523/JNEUROSCI.17-01-00171.1997 PMC6793699

[4] Kira J. Autoimmunity in neuromyelitis optica and opticospinal multiple sclerosis: Astrocytopathy as a common denominator in demyelinating disorders. J Neurol Sci. 2011;311:69–77. 21962794 10.1016/j.jns.2011.08.043

[5] Lucchinetti CF, Guo Y, Popescu BFGh, Fujihara K, Itoyama Y, Misu T. The pathology of an autoimmune astrocytopathy: Lessons learned from neuromyelitis optica: Autoimmune astrocytopathy. Brain Pathol. 2014;24:83–97. 24345222 10.1111/bpa.12099 PMC3905574

[6] Misu T, Höftberger R, Fujihara K, et al Presence of six different lesion types suggests diverse mechanisms of tissue injury in neuromyelitis optica. Acta Neuropathol. 2013;125:815–827. 23579868 10.1007/s00401-013-1116-7 PMC3661909

[7] Takai Y, Misu T, Suzuki H, et al Staging of astrocytopathy and complement activation in neuromyelitis optica spectrum disorders. Brain. 2021;144:2401–2415. 33711152 10.1093/brain/awab102

[8] Duan T, Verkman AS. Experimental animal models of aquaporin-4-IgG-seropositive neuromyelitis optica spectrum disorders: Progress and shortcomings. Brain Pathol. 2020;30:13–25. 31587392 10.1111/bpa.12793 PMC7034663

[9] Howe CL, Kaptzan T, Magaña SM, Ayers-Ringler JR, LaFrance-Corey RG, Lucchinetti CF. Neuromyelitis optica IgG stimulates an immunological response in rat astrocyte cultures. Glia. 2014;62:692–708. 24492996 10.1002/glia.22635 PMC5392242

[10] Marignier R, Nicolle A, Watrin C, et al Oligodendrocytes are damaged by neuromyelitis optica immunoglobulin G via astrocyte injury. Brain. 2010;133:2578–2591. 20688809 10.1093/brain/awq177

[11] Marignier R, Ruiz A, Cavagna S, et al Neuromyelitis optica study model based on chronic infusion of autoantibodies in rat cerebrospinal fluid. J Neuroinflammation. 2016;13:111. 27193196 10.1186/s12974-016-0577-8 PMC4872335

[12] Richard C, Ruiz A, Cavagna S, et al Connexins in neuromyelitis optica: A link between astrocytopathy and demyelination. Brain. 2020;143:2721–2732 . 32889550 10.1093/brain/awaa227

[13] Del Bigio MR. Ependymal cells: Biology and pathology. Acta Neuropathol. 2010;119:55–73. 20024659 10.1007/s00401-009-0624-y

[14] Meunier A, Sawamoto K, Spassky N. Chapter 42 - ependyma. In: Rubenstein J Rakic P Chen B and Kwan KY, eds. Patterning and cell type specification in the developing CNS and PNS (second edition). Academic Press; 2020:1021–1036.

[15] Zhang J, Chandrasekaran G, Li W, et al Wnt-PLC-IP3-connexin-Ca2 + axis maintains ependymal motile cilia in zebrafish spinal cord. Nat Commun. 2020;11:1860. 32312952 10.1038/s41467-020-15248-2 PMC7170879

[16] Pourabdolhossein F, Gil-Perotín S, Garcia-Belda P, et al Inflammatory demyelination induces ependymal modifications concomitant to activation of adult (SVZ) stem cell proliferation. Glia. 2017;65:756–772. 28191668 10.1002/glia.23124

[17] Young CC, van der Harg JM, Lewis NJ, Brooks KJ, Buchan AM, Szele FG. Ependymal ciliary dysfunction and reactive astrocytosis in a reorganized subventricular zone after stroke. Cerebral Cortex. 2013;23:647–659. 22414771 10.1093/cercor/bhs049 PMC3563342

[18] Banker P, Sonni S, Kister I, Loh JP, Lui YW. Pencil-thin ependymal enhancement in neuromyelitis optica spectrum disorders. Multi Scler. 2012;18:1050–1053. 10.1177/1352458511431730 22183933

[19] long Y, Chen M, Zhang B, et al Brain gadolinium enhancement along the ventricular and leptomeningeal regions in patients with aquaporin-4 antibodies in cerebral spinal fluid. J Neuroimmunol. 2014;269:62–67. 24582827 10.1016/j.jneuroim.2014.02.006

[20] Guo Y, Weigand SD, Popescu BF, et al Pathogenic implications of cerebrospinal fluid barrier pathology in neuromyelitis optica. Acta Neuropathol. 2017;133:597–612. 28184993 10.1007/s00401-017-1682-1 PMC5348570

[21] Tritschler F, Murín R, Birk B, et al Thrombin causes the enrichment of rat brain primary cultures with ependymal cells via protease-activated receptor 1. Neurochem Res. 2007;32:1028–1035. 17401674 10.1007/s11064-006-9267-8

[22] Weibel M, Pettmann B, Artault J-C, Sensenbrenner M, Labourdette G. Primary culture of rat ependymal cells in serum-free defined medium. Brain Res. 1986;25:199–209. 10.1016/s0006-8993(86)80228-1 3955370

[23] Mirzadeh Z, Doetsch F, Sawamoto K, Wichterle H, Alvarez-Buylla A. The subventricular zone en-face: Wholemount staining and ependymal flow. J Vis Exp. 2010; 39: 1938. 10.3791/1938 PMC3144601 20461052

[24] Martin M. Cutadapt removes adapter sequences from high–throughput sequencing reads. *EMBnet Journal*. 2011:10-12.

[25] Bray NL, Pimentel H, Melsted P, Pachter L. Near-optimal probabilistic RNA-seq quantification. Nat Biotechnol. 2016;34:525–527. 27043002 10.1038/nbt.3519

[26] Love MI, Huber W, Anders S. Moderated estimation of fold change and dispersion for RNA-seq data with DESeq2. Genome Biol. 2014;15:550. 25516281 10.1186/s13059-014-0550-8 PMC4302049

[27] Subramanian A, Tamayo P, Mootha VK, et al Gene set enrichment analysis: A knowledge-based approach for interpreting genome-wide expression profiles. Proc Natl Acad Sci U S A. 2005;102:15545–15550. 16199517 10.1073/pnas.0506580102 PMC1239896

[28] Shannon P, Markiel A, Ozier O, et al Cytoscape: A software environment for integrated models of biomolecular interaction networks. Genome Res. 2003;13:2498–2504. 14597658 10.1101/gr.1239303 PMC403769

[29] Merico D, Isserlin R, Stueker O, Emili A, Bader GD. Enrichment map: A network-based method for gene-set enrichment visualization and interpretation. PLoS One. 2010;5:e13984. 21085593 10.1371/journal.pone.0013984 PMC2981572

[30] Morales A, Bonnet C, Bourgoin N, et al Unexpected expression of orexin-B in basal conditions and increased levels in the adult rat hippocampus during pilocarpine-induced epileptogenesis. Brain Res. 2006;1109:164–175. 16904080 10.1016/j.brainres.2006.06.075

[31] Core Team R. R: A language and environment for statistical computing. R Foundation for Statistical Computing; 2022.

[32] Kuznetsova A, Brockhoff PB, Christensen RHB. Lmertest package: Tests in linear mixed effects models. J Stat Softw. 2017;82:1–26.

[33] Jammalamadaka SR, Sengupta A. Topics in circular statistics. World Scientific. 2001.

[34] Mirzadeh Z, Merkle FT, Soriano-Navarro M, Garcia-Verdugo JM, Alvarez-Buylla A. Neural stem cells confer unique pinwheel architecture to the ventricular surface in neurogenic regions of the adult brain. Cell Stem Cell. 2008;3:265–278. 18786414 10.1016/j.stem.2008.07.004 PMC2613692

[35] Spassky N, Meunier A. The development and functions of multiciliated epithelia. Nat Rev Mol Cell Biol. 2017;18:423–436. 28400610 10.1038/nrm.2017.21

[36] Takagishi M, Sawada M, Ohata S, et al Daple coordinates planar polarized microtubule dynamics in ependymal cells and contributes to hydrocephalus. Cell Rep. 2017;20:960–972. 28746879 10.1016/j.celrep.2017.06.089

[37] Gómez-Pinedo U, García-Ávila Y, Gallego-Villarejo L, et al Sera from patients with NMOSD reduce the differentiation capacity of precursor cells in the central nervous system. Int J Mol Sci. 2021;22:5192. 34068922 10.3390/ijms22105192 PMC8155872

[38] Sawamoto K. New neurons follow the flow of cerebrospinal fluid in the adult brain. Science. 2006;311:629–632. 16410488 10.1126/science.1119133

[39] Hinson SR, Romero MF, Popescu BFG, et al Molecular outcomes of neuromyelitis optica (NMO)-IgG binding to aquaporin-4 in astrocytes. Proc Natal Acad Sci U S A. 2012;109:1245–1250. 10.1073/pnas.1109980108 PMC3268278 22128336

[40] Verkman AS, Ratelade J, Rossi A, Zhang H, Tradtrantip L. Aquaporin-4: Orthogonal array assembly, CNS functions, and role in neuromyelitis optica. Acta Pharmacol Sin. 2011;32:702–710. 21552296 10.1038/aps.2011.27 PMC3601948

[41] Asgari N, Khorooshi R, Lillevang ST, Owens T. Complement-dependent pathogenicity of brain-specific antibodies in cerebrospinal fluid. J Neuroimmunol. 2013;254:76–82. 23031833 10.1016/j.jneuroim.2012.09.010

[42] Mack A, Neuhaus J, Wolburg H. Relationship between orthogonal arrays of particles and tight junctions as demonstrated in cells of the ventricular wall of the rat brain. Cell Tissue Res. 1987;248:619–625. 3607852 10.1007/BF00216492

[43] Diamond B, Huerta PT, Mina-Osorio P, Kowal C, Volpe BT. Losing your nerves? Maybe it’s the antibodies. Nat Rev Immunol. 2009;9:449–456. 19424277 10.1038/nri2529 PMC2783680

[44] Mahuzier A, Shihavuddin A, Fournier C, et al Ependymal cilia beating induces an actin network to protect centrioles against shear stress. Nat Commun. 2018;9:2279. 29891944 10.1038/s41467-018-04676-w PMC5996024

[45] den Hollander AI, Roepman R, Koenekoop RK, Cremers FPM. Leber congenital amaurosis: Genes, proteins and disease mechanisms. Prog Retin Eye Res. 2008;27:391–419. 18632300 10.1016/j.preteyeres.2008.05.003

[46] Qu Z, Yimer TA, Xie S, et al Knocking out lca5 in zebrafish causes cone-rod dystrophy due to impaired outer segment protein trafficking. Biochim Biophys Acta Mol Basis Dis. 2019;1865:2694–2705. 31348989 10.1016/j.bbadis.2019.07.009

[47] Blacque OE, Cevik S, Kaplan OI. Intraflagellar transport: From molecular characterisation to mechanism. Front Biosci. 2008;13:2633–2652. 17981739 10.2741/2871

[48] Pittock SJ, Weinshenker BG, Lucchinetti CF, Wingerchuk DM, Corboy JR, Lennon VA. Neuromyelitis optica brain lesions localized at sites of high aquaporin 4 expression. Arch Neurol. 2006;63:964–968. 16831965 10.1001/archneur.63.7.964

[49] Zywitza V, Misios A, Bunatyan L, Willnow TE, Rajewsky N. Single-cell transcriptomics characterizes cell types in the subventricular zone and uncovers molecular defects impairing adult neurogenesis. Cell Rep. 2018;25:2457–2469.e8. 30485812 10.1016/j.celrep.2018.11.003

[50] Belenguer G, Duart-Abadia P, Jordán-Pla A. Adult neural stem cells are alerted by systemic inflammation through TNF-α receptor signaling. Cell Stem Cell 2021;28:285–299. 33207218 10.1016/j.stem.2020.10.016

[51] Whish S, Dziegielewska KM, MÃ¸llgÃ¥rd Kjeld, et al The inner CSF-brain barrier: Developmentally controlled access to the brain via intercellular junctions. Front Neurosci. 2015;9:16. 25729345 10.3389/fnins.2015.00016 PMC4325900

[52] Cobo-Calvo A, Ruiz A, Richard C, et al Purified IgG from aquaporin-4 neuromyelitis optica spectrum disorder patients alters blood-brain barrier permeability. PLoS One. 2020;15:e0238301. 32881954 10.1371/journal.pone.0238301 PMC7470361

[53] Alvarez E, Piccio L, Mikesell RJ, et al CXCL13 is a biomarker of inflammation in multiple sclerosis, neuromyelitis optica, and other neurological conditions. Mult Scler. 2013;19:1204–1208. 23322500 10.1177/1352458512473362 PMC3959125

[54] Koper OM, Kamińska J, Grygorczuk S, Zajkowska J, Kemona H. CXCL9 concentrations in cerebrospinal fluid and serum of patients with tick-borne encephalitis. Arch Med Sci. 2018;14:313–320. 29593804 10.5114/aoms.2016.58667 PMC5868655

[55] Sato DK, Callegaro D, Jorge H, et al Cerebrospinal fluid aquaporin-4 antibody levels in neuromyelitis optica attacks. Ann Neurol. 2014;76:305–309. 24977390 10.1002/ana.24208 PMC4173125

[56] Schneider R, Bellenberg B, Kleiter I, et al Cervical cord and ventricle affection in neuromyelitis optica. Acta Neurol Scand. 2017;135:324–331. 27098675 10.1111/ane.12601

[57] Ducloyer J-B, Marignier R, Wiertlewski S, Lebranchu P. Optic neuritis classification in 2021. Eur J Ophthalmol. 2021:11206721211028050. 10.1177/11206721211028050 34218696

[58] Shah PT, Stratton JA, Stykel MG, et al Single-cell transcriptomics and fate mapping of ependymal cells reveals an absence of neural stem cell function. Cell. 2018;173:1045–1057.e9. 29727663 10.1016/j.cell.2018.03.063

[59] Spassky N. Adult ependymal cells are postmitotic and are derived from radial glial cells during embryogenesis. J Neurosci. 2005;25:10–18. 15634762 10.1523/JNEUROSCI.1108-04.2005 PMC6725217

