# Supplementary material for: Ependyma: a new target for autoantibodies in neuromyelitis optica?
Source: Brain Commun. 2022 Nov 30;4(6):fcac307. doi: 10.1093/braincomms/fcac307 (PMC9897195; doi:10.1093/braincomms/fcac307)
Supplement: fcac307_Supplementary_Data [file fcac307_Supplementary_Data.zip › Supplementary_material_and_Supplementary_figures.docx]

# Supplementary material


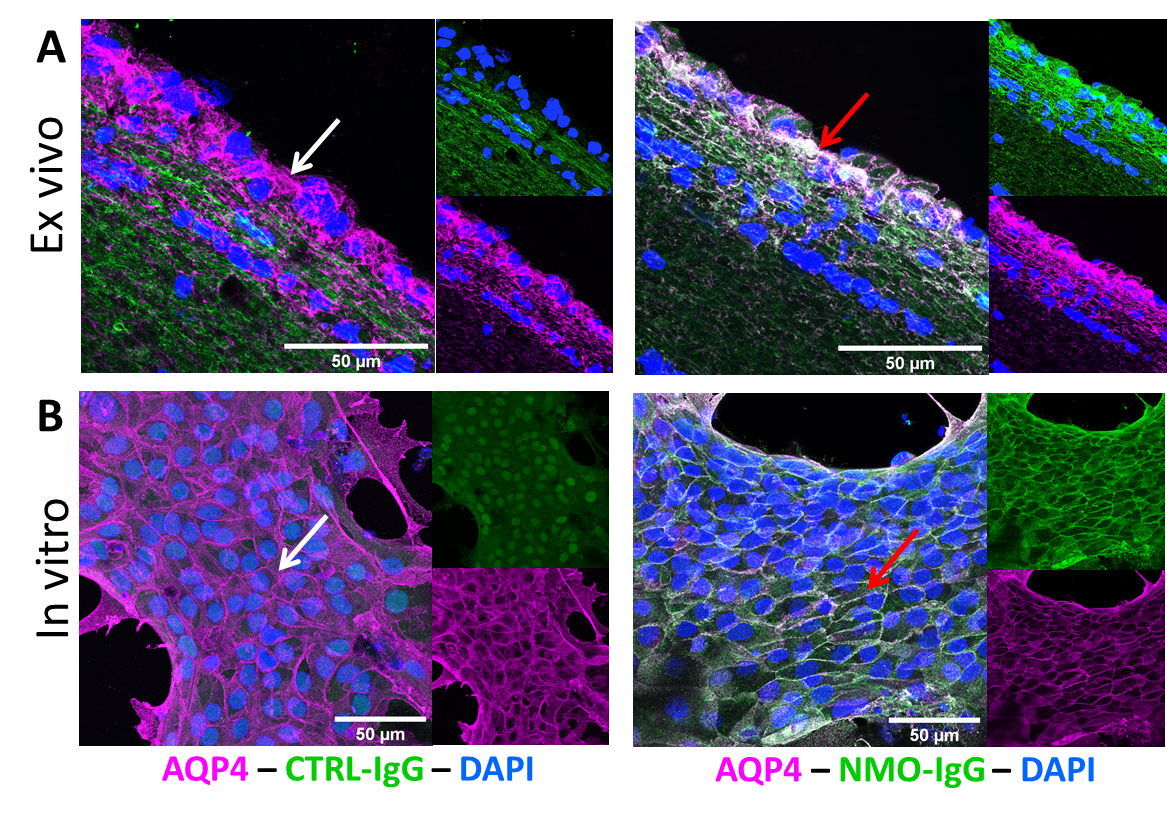


**Supplementary figure 1 Purified IgG from NMO patients bind to ependymal AQP4 ex vivo and in vitro.** Confocal images showing no colocalization of CTRL-IgG (green) with AQP4 (magenta) (white arrows), and colocalization of NMO-IgG (green) with AQP4 (magenta) (red arrows) on (**A**) healthy rat lateral ventricles (x63) and (**B**) ependymal cell primary cultures (x40) post fixation.

**
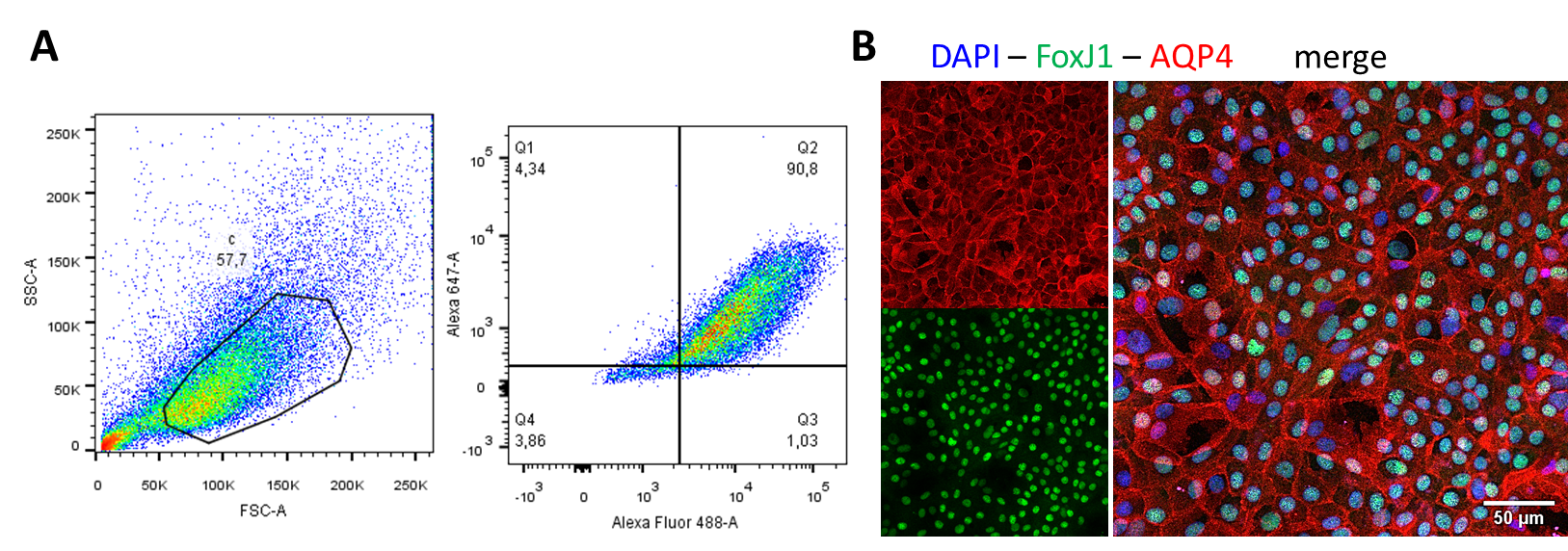
**

**Supplementary figure 2 Proportion of FoxJ1^+^ cells in primary ependymal cell cultures.** (**A**) Ependymal cells co-stained for FoxJ1 (Alexa 647) and AQP4 (Alexa 488) were cell sorted by flow cytometry after no treatment (*n* = 1). (**B**) Epifluorescence images of primary culture of ependymal cells labeled for FoxJ1 (green), AQP4 (red) and DAPI (blue).


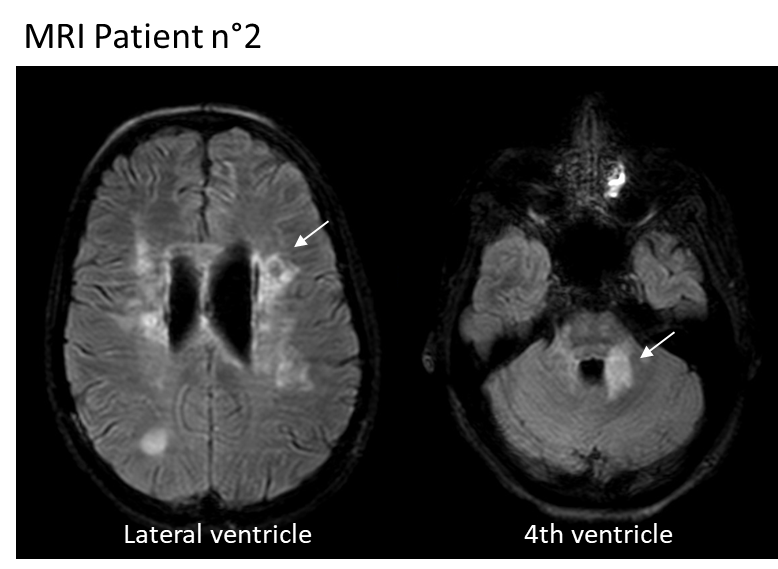


**Supplementary Figure 3 Periependymal lesion on MRI from patient n°2**. Brain MRI axial T2-weighted fluid-attenuated inversion recovery (FLAIR) images were realized prior to plasma exchange and revealed periependymal lesions around the lateral ventricles and the 4^th^ ventricle (white arrow).


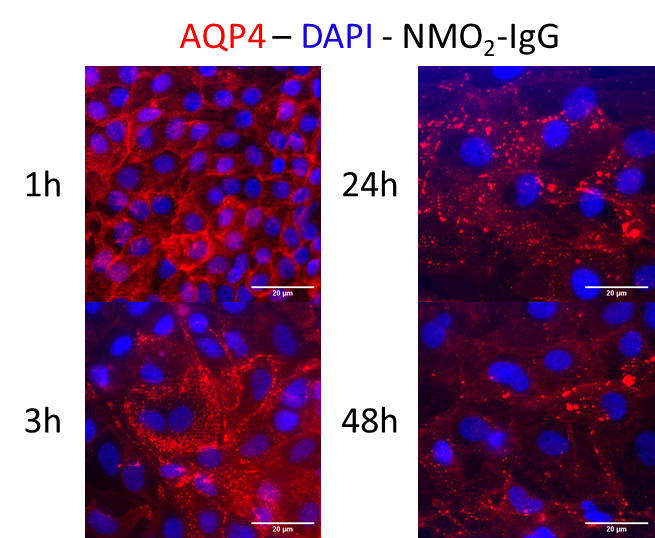


**Supplementary figure 4 Kinetic of NMO-IgG induced AQP4 agglomeration.** Primary cultures were treated during 1, 3, 24 and 48 hours with NMO_2_-IgG and stained for AQP4 (red) and DAPI (blue).


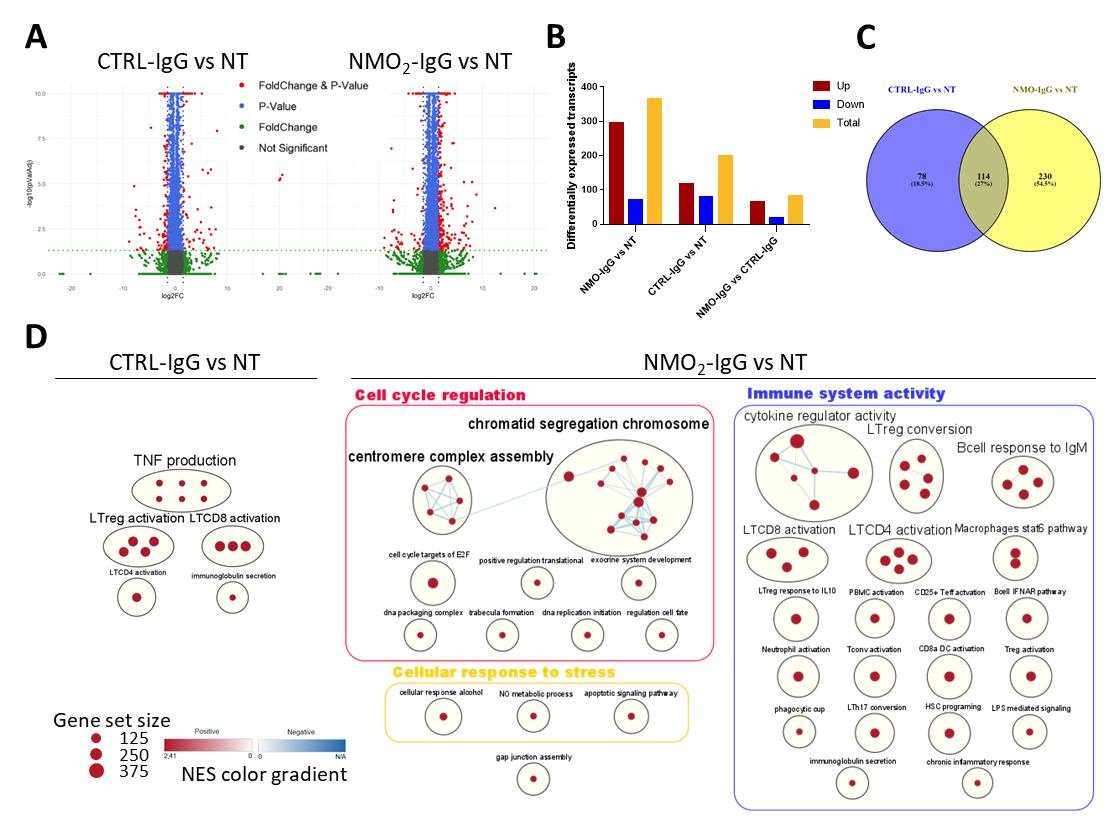


**Supplementary figure 5 NMO-IgG changed the transcriptomic profile of ependymal cells.** (**A**) Transcriptomic differential analysis between CTRL-IgG vs NT and NMO_2_-IgG vs NT ependymal cells from primary cultures represented as volcano-plots (*n* = 3 cultures). Red dots represent transcripts that are significantly upregulated (right) or downregulated (left) (statistical criteria: |log2FC*|* ≥ 1.5 and Wald’s test adjusted *P_adj_* ≤ 0.05). (**B**) Histogram showing the number of transcripts differentially expressed in CTRL-IgG vs NT and NMO_2_-IgG vs NT conditions. (**C**) Venn diagram showing the number of genes shared by the differential expression of CTRL-IgG vs NT and NMO_2_-IgG vs NT conditions. (**D**) Enrichment map of the GSEA of CTRL-IgG vs NT and NMO_2_-IgG vs NT. Cut-offs applied: gene set size between 500 and 15 genes and *FDR q-value* < 0.05. Dot size: gene set size. Dot color: normalized enrichment score (NES). Blue edge size: number of common genes. Nodes with high similarity were clustered using the AutoAnnotate app of Cytoscape.


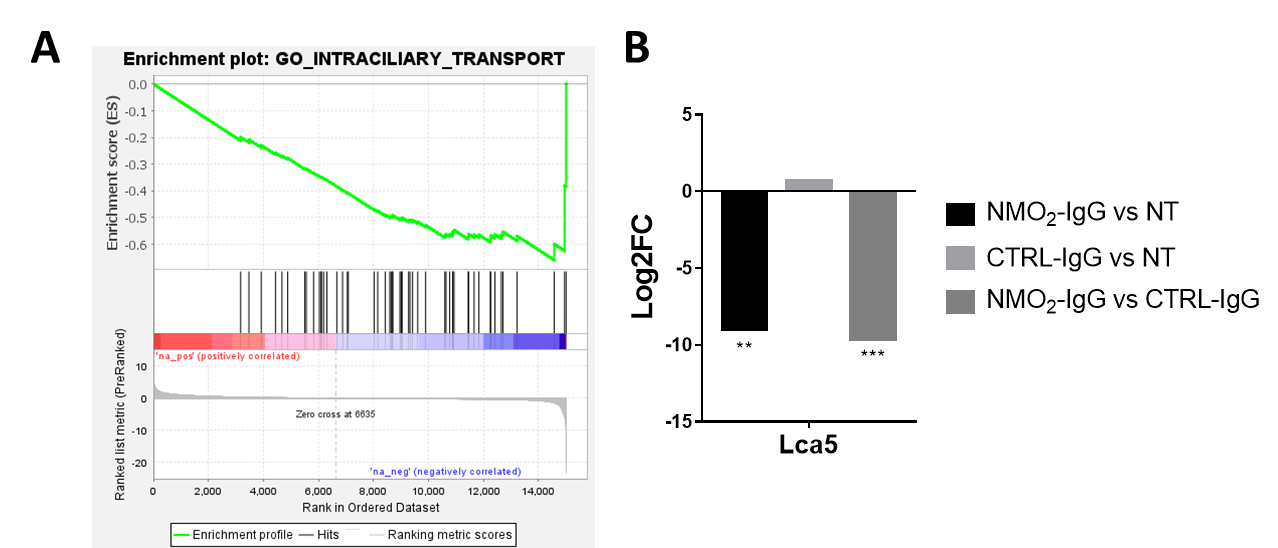


**Supplementary figure 6 NMO-IgG downregulated intraciliary transport.** (**A**) Enrichment plot of the gene set GO_Intraciliary_Transport downregulated in NMO_2_-IgG vs NT (Normalized Enrichment Score (NES) = -1.78, *P* = 0.002, *FDR q-value* = 0.631). (**B**) Histogram of the Log2FC for the LCA5 transcript from the RNAseq data in NT vs NMO_2_-IgG (*P_adj_* < 0.01), NT vs CTRL-IgG (non-significant) and NMO_2_-IgG vs CTRL-IgG (*P_adj_* < 0.001) comparisons.

# Materiel and methods

## Binding of purified IgG from NMO patients on AQP4 expressed by ependymal cells

To test the ability of purified IgG from NMO patients to bind AQP4, fixed ependymal cells from primary cultures or from brain slices of adult Sprague Dawley rat brain were incubated with NMO-IgG or CTRL-IgG during 24 hours at 4°C. After washing steps (see the immunolabeling part in the main document and table 1 for more details), samples were incubated with commercial anti-AQP4 during 1h at 37°C. After washing steps, samples were incubated with anti-rabbit AF555 secondary antibody and with donkey biotin SP-conjugated anti-human IgG for one hour at room temperature. After washing steps, samples were incubated with streptavidin AF488 during three hours at room temperature. After washing steps and nuclei staining with DAPI, slides were mounted and analyzed with an epifluorescent microscope.

## Flow cytometry

Ependymal cells from primary cultures were detached with accutase and cell viability was determined using trypan blue (more than 90% of cells were viable). Cells were centrifugated five minutes at 1400 rpm and suspended in PBS with 8% normal goat serum for saturation during 20 minutes at 4°C. Cells were fixed with PFA 1% during 15 minutes at room temperature. Then, cells were permeabilized with a PBS saponin solution at 0.5% during 20 minutes at room temperature. After permeabilization, cells were incubated with rabbit anti-AQP4 and mouse anti-FoxJ1 primary antibodies (Supplementary Table 1) diluted in PBS saponin 0.05% during 20 minutes at 4°C. After two washes with PBS saponin 0.05%, cells were incubated with biotin conjugated goat anti-mouse and goat anti-rabbit AF488 for 20 minutes at 4°C. After two washes with PBS saponin 0.05%, cells were incubated with streptavidin conjugated AF647 during 20 minutes at 4°C. After centrifugation and resuspension, the percentage of ependymal cells defined as double positive from AQP4 and FoxJ1 was measured by flow cytometry with a FACS Canto II (Beckton-Dickinson, San Diego, CA, USA) equipped with 3 lasers (488, 633, 405 nm) using the BD FACS-DivaTM software and FlowJo software (version 10 by Flowjo LLC, OR, USA).
