# Supplementary material for: Ependyma: a new target for autoantibodies in neuromyelitis optica?
Source: Brain Commun. 2022 Nov 30;4(6):fcac307. doi: 10.1093/braincomms/fcac307 (PMC9897195; doi:10.1093/braincomms/fcac307)
Supplement: fcac307_Supplementary_Data [file fcac307_Supplementary_Data.zip › Supplementary_material_Mixed-models_R_scripts.docx]

Bigotte et al, Mixed models

Antoine & Maxime

2022-07-22

# librairies
library(ggplot2)
library(lme4)
library(lmerTest)
library(car)
library(dplyr)

#### Bead speed

# import et preparation des données

data <- read.table("Bead_speedV3.csv", h = T, sep = ",")
data$TTT <- relevel(as.factor(data$TTT), "NT")

#attention à ce que la variable qui sera l'effet aléatoire soit bien catégorielle!

data$Explant <- as.factor(paste0(data$TTT, data$Explant))
data$Rat <- as.factor(data$Rat)

# representation graphique
ggplot(data) +
 geom_boxplot (aes(y = Speed, x = TTT, fill = TTT), alpha = 2/3)+
 geom_boxplot (aes(y = Speed, x = TTT, group = Explant, color = Rat), linetype = 1, alpha = 1/3) +
 theme_classic ()


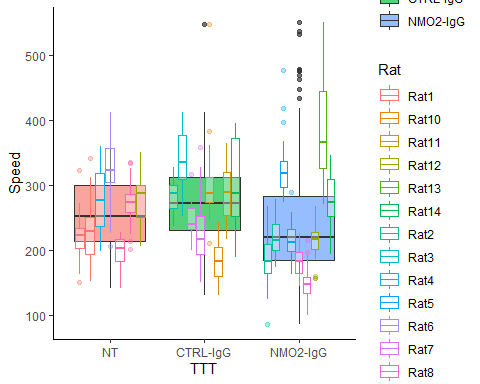


# modeles à effets mixtes avec le rat en effet imbriqué
modmix1 <-lmerTest::lmer(Speed ~ TTT + (1|Rat/Explant), data = data)
summary(modmix1)

## Linear mixed model fit by REML. t-tests use Satterthwaite's method [
## lmerModLmerTest]
## Formula: Speed ~ TTT + (1 | Rat/Explant)
## Data: data
##
## REML criterion at convergence: 7474.5
##
## Scaled residuals:
## Min 1Q Median 3Q Max
## -2.8111 -0.5725 -0.0291 0.5218 6.0317
##
## Random effects:
## Groups Name Variance Std.Dev.
## Explant:Rat (Intercept) 2165 46.53
## Rat (Intercept) 1554 39.42
## Residual 1710 41.36
## Number of obs: 720, groups: Explant:Rat, 24; Rat, 14
##
## Fixed effects:
## Estimate Std. Error df t value Pr(>|t|)
## (Intercept) 265.102 25.075 14.041 10.573 4.54e-08 ***
## TTTCTRL-IgG 8.145 34.791 13.589 0.234 0.818
## TTTNMO2-IgG -22.704 32.328 18.036 -0.702 0.491
## ---
## Signif. codes: 0 '***' 0.001 '**' 0.01 '*' 0.05 '.' 0.1 ' ' 1
##
## Correlation of Fixed Effects:
## (Intr) TTTCTR
## TTTCTRL-IgG -0.717
## TTTNMO2-IgG -0.725 0.554

# vérifications des hypothéses : normalité des résidus et des effets aléatoires
qqPlot(resid(modmix1))


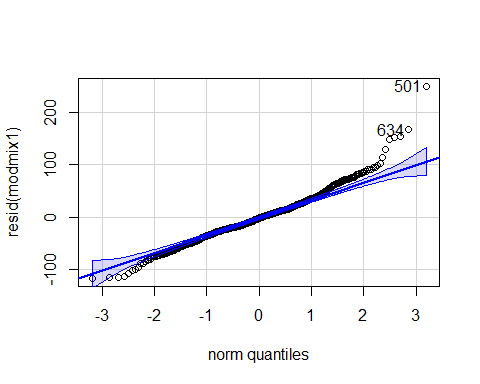


## [1] 501 634

qqPlot(ranef(modmix1)$"Explant:Rat"[,1])


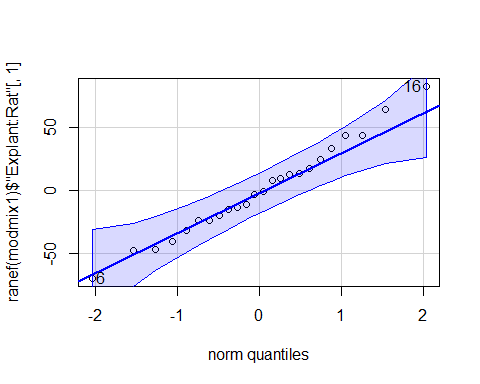


## [1] 16 6

qqPlot(ranef(modmix1)$`Rat`[,1])


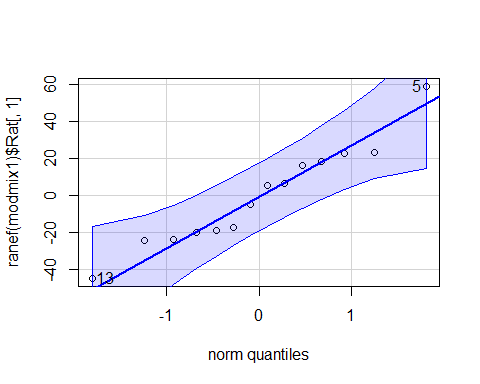


## [1] 5 13

#### Directionnaliy

data2 <- read.table("DirectionalityV3.csv", h = T, sep = ",")

data2$TTT <- relevel(as.factor(data2$TTT), "NT")
data2$Explant <- as.factor(data2$Explant)
data2$Rat <- as.factor(data2$Rat)

# representation graphique

ggplot(data2) +
 geom_boxplot (aes(y = Directionality, x = TTT, fill = TTT), alpha = 2/3)+
 geom_boxplot (aes(y = Directionality, x = TTT, group = Explant, color = Rat), linetype = 1, alpha = 1/3) +
 theme_classic ()


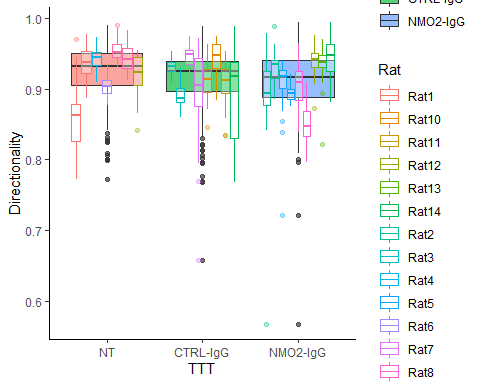


# modèles à effets mixtes avec le rat en effet imbriqué
modmix2 <-lmerTest::lmer(Directionality ~ TTT + (1|Rat/Explant), data = data2)

## boundary (singular) fit: see ?isSingular

summary(modmix2)

## Linear mixed model fit by REML. t-tests use Satterthwaite's method [
## lmerModLmerTest]
## Formula: Directionality ~ TTT + (1 | Rat/Explant)
## Data: data2
##
## REML criterion at convergence: -2743.3
##
## Scaled residuals:
## Min 1Q Median 3Q Max
## -9.7196 -0.3921 0.0786 0.5002 3.1829
##
## Random effects:
## Groups Name Variance Std.Dev.
## Explant:Rat (Intercept) 7.847e-04 2.801e-02
## Rat (Intercept) 2.607e-18 1.615e-09
## Residual 1.140e-03 3.376e-02
## Number of obs: 720, groups: Explant:Rat, 24; Rat, 14
##
## Fixed effects:
## Estimate Std. Error df t value Pr(>|t|)
## (Intercept) 0.92351 0.01084 21.00000 85.186 <2e-16 ***
## TTTCTRL-IgG -0.00924 0.01485 21.00000 -0.622 0.540
## TTTNMO2-IgG -0.01311 0.01446 21.00000 -0.907 0.375
## ---
## Signif. codes: 0 '***' 0.001 '**' 0.01 '*' 0.05 '.' 0.1 ' ' 1
##
## Correlation of Fixed Effects:
## (Intr) TTTCTR
## TTTCTRL-IgG -0.730
## TTTNMO2-IgG -0.750 0.548
## optimizer (nloptwrap) convergence code: 0 (OK)
## boundary (singular) fit: see ?isSingular

# vérifications des hypothéses : normalité des résidus et des effets aléatoires
qqPlot(resid(modmix2))


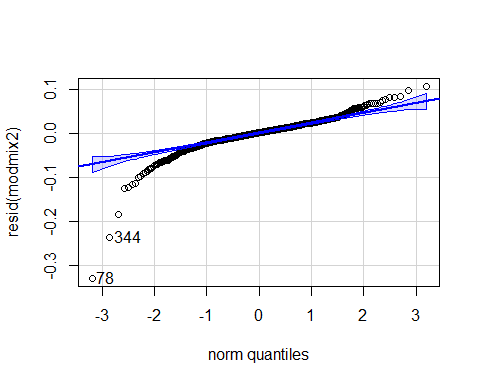


## [1] 78 344

qqPlot(ranef(modmix2)$"Explant:Rat"[,1])


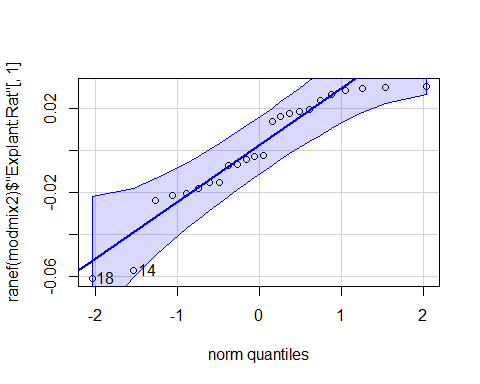


## [1] 18 14

qqPlot(ranef(modmix2)$`Rat`[,1])


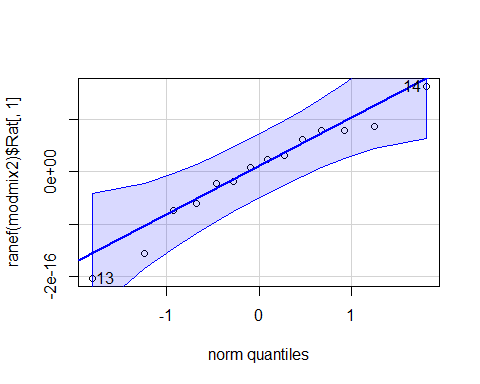


## [1] 13 14

#### %AQP4 agglomération

# import et preparation des données
data3 <- read.table("%AQP4agglomeration.csv", h = T, sep = ",")
data3$TTT <- relevel(as.factor(data3$TTT), "NT")
data3$Culture <- as.factor(data3$Culture)
data3$Puit <- as.factor(paste0(data3$Culture, data3$TTT))


# representation graphique
ggplot(data3) +
 geom_boxplot (aes(y = X.agglo, x = TTT, fill = TTT), alpha = 2/3) +
 geom_boxplot (aes(y = X.agglo, x = TTT, group = Puit, color = Culture), linetype = 1, alpha = 1/3) +
 theme_classic ()


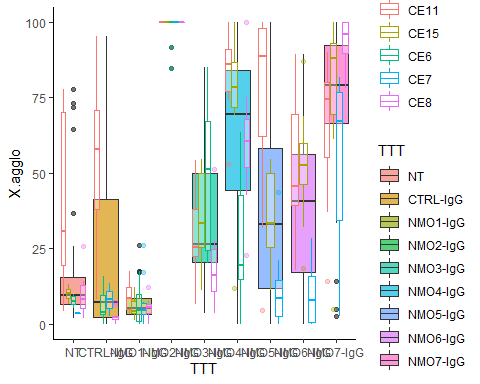


# modeles à effets mixtes
modmix3 <-lmerTest::lmer(X.agglo ~ TTT + Culture + (1|Puit), data = data3)
summary(modmix3)

## Linear mixed model fit by REML. t-tests use Satterthwaite's method [
## lmerModLmerTest]
## Formula: X.agglo ~ TTT + Culture + (1 | Puit)
## Data: data3
##
## REML criterion at convergence: 2882.2
##
## Scaled residuals:
## Min 1Q Median 3Q Max
## -4.3640 -0.3085 -0.0102 0.4074 2.6528
##
## Random effects:
## Groups Name Variance Std.Dev.
## Puit (Intercept) 207.5 14.40
## Residual 271.8 16.49
## Number of obs: 343, groups: Puit, 37
##
## Fixed effects:
## Estimate Std. Error df t value Pr(>|t|)
## (Intercept) 28.705 8.653 26.182 3.317 0.002673 **
## TTTCTRL-IgG 5.196 11.007 28.237 0.472 0.640491
## TTTNMO1-IgG -7.103 10.042 26.054 -0.707 0.485610
## TTTNMO2-IgG 85.674 10.042 26.054 8.532 5.09e-09 ***
## TTTNMO3-IgG 16.153 10.623 25.356 1.521 0.140751
## TTTNMO4-IgG 44.247 10.623 25.356 4.165 0.000316 ***
## TTTNMO5-IgG 24.490 11.779 25.688 2.079 0.047735 *
## TTTNMO6-IgG 21.732 11.779 25.688 1.845 0.076596 .
## TTTNMO7-IgG 57.801 10.722 25.804 5.391 1.23e-05 ***
## CultureCE15 -10.070 7.506 23.774 -1.342 0.192413
## CultureCE6 -20.237 8.420 24.677 -2.404 0.024085 *
## CultureCE7 -27.849 8.166 26.407 -3.410 0.002099 **
## CultureCE8 -16.101 7.890 23.741 -2.041 0.052546 .
## ---
## Signif. codes: 0 '***' 0.001 '**' 0.01 '*' 0.05 '.' 0.1 ' ' 1

##
## Correlation matrix not shown by default, as p = 13 > 12.
## Use print(x, correlation=TRUE) or
## vcov(x) if you need it

# vérifications des hypothéses : normalité des résidus et des effets aléatoires
qqPlot(resid(modmix3))


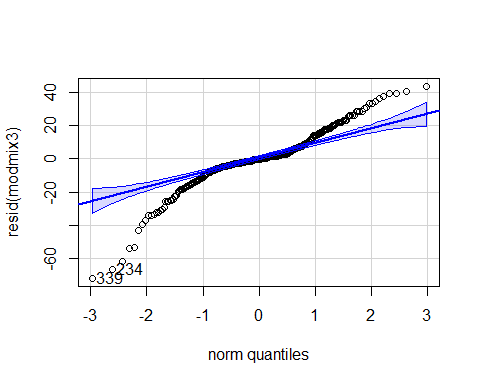


## [1] 339 234

qqPlot(ranef(modmix3)$Puit[,1])


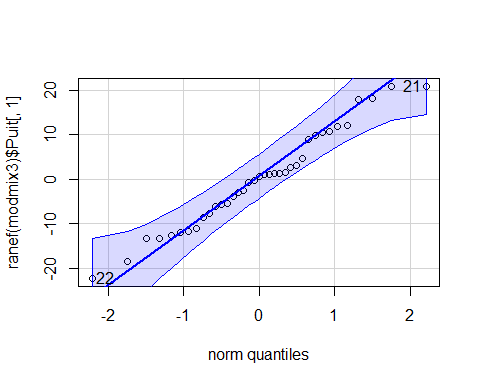


## [1] 22 21

#### B cells

# import et preparation des données
data4 <- read.table("B cells.csv", h = T, sep = "," , dec = ",")
data4$TTT <- relevel(as.factor(data4$TTT), "NT")
data4$Explant <- as.factor(data4$Explant)
data4$Rat <- as.factor(data4$Rat)

# representation graphique
ggplot(data4) +
 geom_boxplot (aes(y = Number.of.B.cells, x = TTT, fill = TTT), alpha = 2/3) +
 geom_boxplot (aes(y = Number.of.B.cells, x = TTT, group = Explant, color = Rat), linetype = 1, alpha = 1/3) +
 theme_classic ()


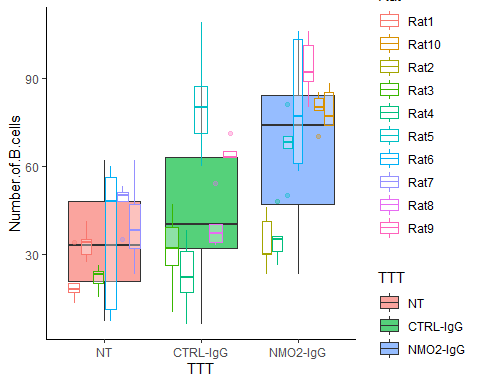


# modeles à effets mixtes
modmix4 <-lmerTest::lmer(Number.of.B.cells ~ TTT + (1|Rat/Explant), data = data4)
summary(modmix4)

## Linear mixed model fit by REML. t-tests use Satterthwaite's method [
## lmerModLmerTest]
## Formula: Number.of.B.cells ~ TTT + (1 | Rat/Explant)
## Data: data4
##
## REML criterion at convergence: 729.6
##
## Scaled residuals:
## Min 1Q Median 3Q Max
## -2.46668 -0.48200 -0.08227 0.50170 2.58433
##
## Random effects:
## Groups Name Variance Std.Dev.
## Explant:Rat (Intercept) 84.73 9.205
## Rat (Intercept) 312.29 17.672
## Residual 154.24 12.420
## Number of obs: 90, groups: Explant:Rat, 18; Rat, 10
##
## Fixed effects:
## Estimate Std. Error df t value Pr(>|t|)
## (Intercept) 31.509 8.669 14.268 3.635 0.00263 **
## TTTCTRL-IgG 18.074 9.690 11.283 1.865 0.08836 .
## TTTNMO2-IgG 31.824 9.495 11.989 3.352 0.00577 **
## ---
## Signif. codes: 0 '***' 0.001 '**' 0.01 '*' 0.05 '.' 0.1 ' ' 1
##
## Correlation of Fixed Effects:
## (Intr) TTTCTR
## TTTCTRL-IgG -0.627
## TTTNMO2-IgG -0.661 0.685

# vérifications des hypothéses : normalité des résidus et des effets aléatoires
qqPlot(resid(modmix4))


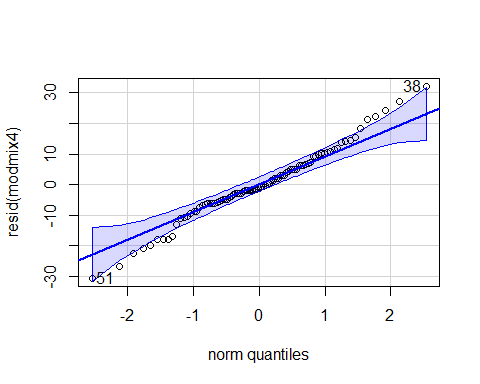


## [1] 38 51

qqPlot(ranef(modmix4)$"Explant:Rat"[,1])


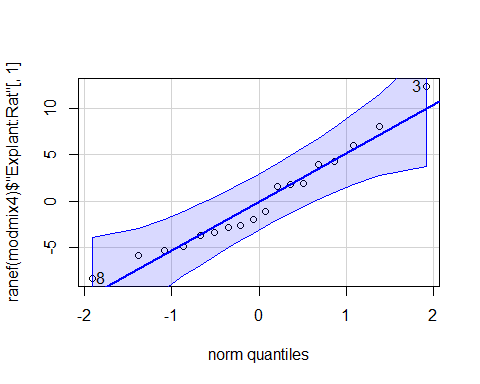


## [1] 3 8

qqPlot(ranef(modmix4)$`Rat`[,1])


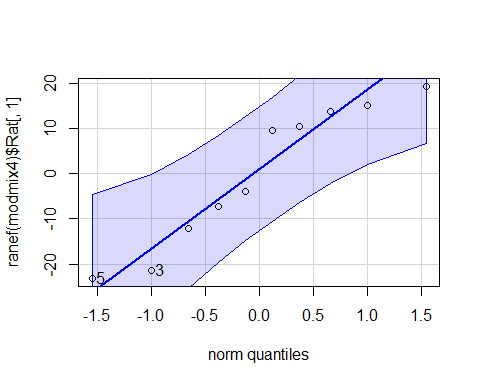


## [1] 5 3

#### Cell area

# import et preparation des données
data5 <- read.table("Cell area.csv", h = T, sep = ",")
data5$TTT <- relevel(as.factor(data5$TTT), "NT")
data5$Explant <- as.factor(data5$Explant)
data5$Rat <- as.factor(data5$Rat)

# representation graphique
ggplot(data5) +
 geom_boxplot (aes(y = Area, x = TTT, fill = TTT), alpha = 2/3) +
 geom_boxplot (aes(y = Area, x = TTT, group = Explant, color = Rat), linetype = 1, alpha = 1/3) +
 theme_classic ()


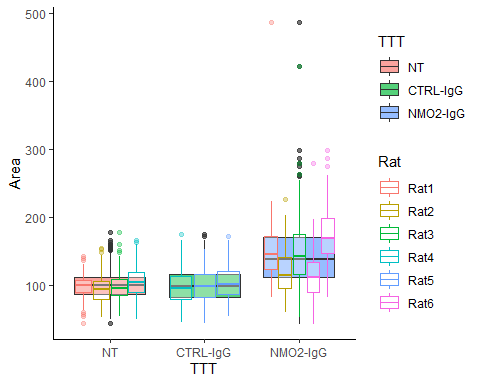


# modeles à effets mixtes
modmix5 <-lmerTest::lmer(Area ~ TTT + (1|Rat/Explant), data = data5)

## boundary (singular) fit: see ?isSingular

summary(modmix5)

## Linear mixed model fit by REML. t-tests use Satterthwaite's method [
## lmerModLmerTest]
## Formula: Area ~ TTT + (1 | Rat/Explant)
## Data: data5
##
## REML criterion at convergence: 14108.3
##
## Scaled residuals:
## Min 1Q Median 3Q Max
## -3.0094 -0.6094 -0.0764 0.5069 10.3610
##
## Random effects:
## Groups Name Variance Std.Dev.
## Explant:Rat (Intercept) 272.1 16.49
## Rat (Intercept) 0.0 0.00
## Residual 1039.0 32.23
## Number of obs: 1440, groups: Explant:Rat, 12; Rat, 6
##
## Fixed effects:
## Estimate Std. Error df t value Pr(>|t|)
## (Intercept) 99.8168 8.4276 9.1570 11.844 7.38e-07 ***
## TTTCTRL-IgG -0.1508 12.8072 8.9736 -0.012 0.99086
## TTTNMO2-IgG 42.9102 11.2977 9.1275 3.798 0.00412 **
## ---
## Signif. codes: 0 '***' 0.001 '**' 0.01 '*' 0.05 '.' 0.1 ' ' 1
##
## Correlation of Fixed Effects:
## (Intr) TTTCTR
## TTTCTRL-IgG -0.658
## TTTNMO2-IgG -0.746 0.491
## optimizer (nloptwrap) convergence code: 0 (OK)
## boundary (singular) fit: see ?isSingular

# vérifications des hypothéses : normalité des résidus et des effets aléatoires
qqPlot(resid(modmix5))


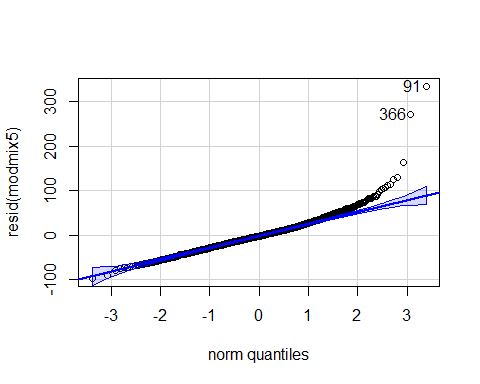


## [1] 91 366

qqPlot(ranef(modmix5)$"Explant:Rat"[,1])


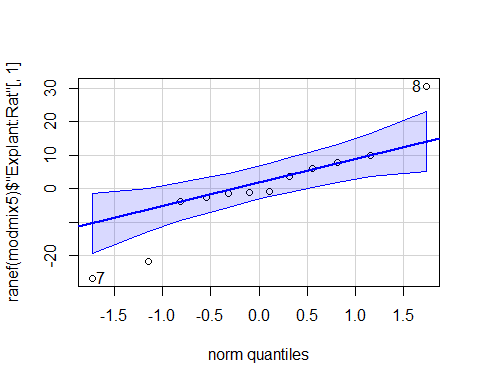


## [1] 8 7

qqPlot(ranef(modmix5)$`Rat`[,1])


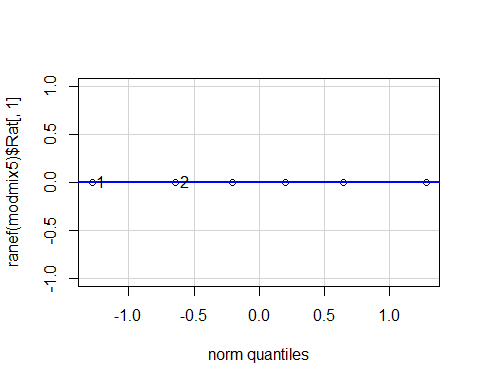


## [1] 1 2

#### Cx43

data6 <- read.table("Cx43particles.csv", h = T, sep = ",")
data6$TTT <- relevel(as.factor(data6$TTT), "NT")
data6$Culture <- as.factor(data6$Culture)
data6$Puit <- as.factor(paste0(data6$Culture, data6$TTT))

# trop peu de conditions de culture pour un effet aléatoire : mise sous forme d'effet fixe
# avec puit en effet aléatoire

# average particle size
# representation graphique
ggplot(data6) +
 geom_boxplot (aes(y = Average_particle_size, x = TTT, fill = TTT), alpha = 2/3) +
 geom_boxplot (aes(y = Average_particle_size, x = TTT, group = Puit, color = Culture), linetype = 1, alpha = 1/3) +
 theme_classic ()


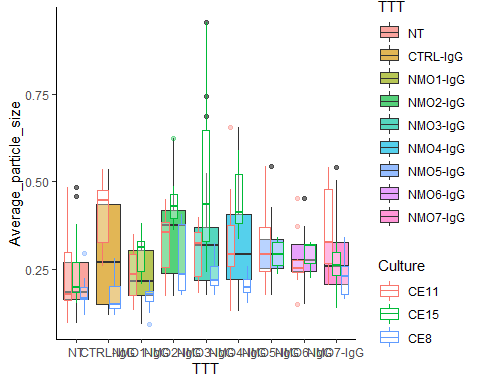


# modeles à effets mixtes
modmix6 <-lmerTest::lmer(Average_particle_size ~ TTT + Culture + (1|Puit), data = data6)
summary(modmix6)

## Linear mixed model fit by REML. t-tests use Satterthwaite's method [
## lmerModLmerTest]
## Formula: Average_particle_size ~ TTT + Culture + (1 | Puit)
## Data: data6
##
## REML criterion at convergence: -376
##
## Scaled residuals:
## Min 1Q Median 3Q Max
## -2.5502 -0.5614 -0.0674 0.4293 5.0427
##
## Random effects:
## Groups Name Variance Std.Dev.
## Puit (Intercept) 0.003045 0.05519
## Residual 0.008867 0.09417
## Number of obs: 240, groups: Puit, 24
##
## Fixed effects:
## Estimate Std. Error df t value Pr(>|t|)
## (Intercept) 0.24285 0.04037 13.00000 6.016 4.33e-05 ***
## TTTCTRL-IgG 0.10794 0.05801 13.00000 1.861 0.08557 .
## TTTNMO1-IgG 0.01427 0.05120 13.00000 0.279 0.78490
## TTTNMO2-IgG 0.12983 0.05120 13.00000 2.536 0.02485 *
## TTTNMO3-IgG 0.12723 0.05120 13.00000 2.485 0.02735 *
## TTTNMO4-IgG 0.10657 0.05120 13.00000 2.081 0.05773 .
## TTTNMO5-IgG 0.04129 0.05809 13.00000 0.711 0.48975
## TTTNMO6-IgG 0.02114 0.05809 13.00000 0.364 0.72176
## TTTNMO7-IgG 0.06360 0.05120 13.00000 1.242 0.23611
## CultureCE15 0.03842 0.03093 13.00000 1.242 0.23604
## CultureCE8 -0.11217 0.03260 13.00000 -3.441 0.00439 **
## ---
## Signif. codes: 0 '***' 0.001 '**' 0.01 '*' 0.05 '.' 0.1 ' ' 1
##
## Correlation of Fixed Effects:
## (Intr) TTTCTR TTTNMO1 TTTNMO2 TTTNMO3 TTTNMO4 TTTNMO5 TTTNMO6
## TTTCTRL-IgG -0.590
## TTTNMO1-IgG -0.634 0.441
## TTTNMO2-IgG -0.634 0.441 0.500
## TTTNMO3-IgG -0.634 0.441 0.500 0.500
## TTTNMO4-IgG -0.634 0.441 0.500 0.500 0.500
## TTTNMO5-IgG -0.597 0.373 0.441 0.441 0.441 0.441
## TTTNMO6-IgG -0.597 0.373 0.441 0.441 0.441 0.441 0.417
## TTTNMO7-IgG -0.634 0.441 0.500 0.500 0.500 0.500 0.441 0.441
## CultureCE15 -0.369 0.138 0.000 0.000 0.000 0.000 -0.010 -0.010
## CultureCE8 -0.377 -0.019 0.000 0.000 0.000 0.000 0.150 0.150
## TTTNMO7 ClCE15
## TTTCTRL-IgG
## TTTNMO1-IgG
## TTTNMO2-IgG
## TTTNMO3-IgG
## TTTNMO4-IgG
## TTTNMO5-IgG
## TTTNMO6-IgG
## TTTNMO7-IgG
## CultureCE15 0.000
## CultureCE8 0.000 0.422

qqPlot(resid(modmix6))


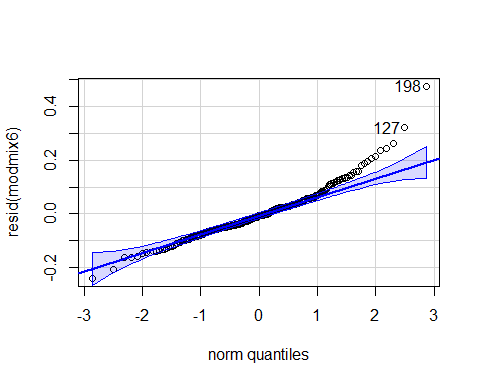


## [1] 198 127

qqPlot(ranef(modmix6)$Puit[,1])


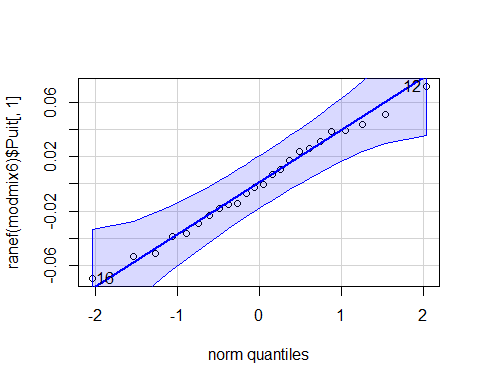


## [1] 12 16

#### ki67

data7 <- read.table("Ki67.csv", h = T, sep = ",")
data7$TTT <- relevel(as.factor(data7$TTT), "NT")
data7$Explant <- as.factor(data7$Explant)
data7$Rat <- as.factor(data7$Rat)

# representation graphique
ggplot(data7) +
 geom_boxplot (aes(y = prop_Ki67, x = TTT, fill = TTT), alpha = 2/3) +
 geom_boxplot (aes(y = prop_Ki67, x = TTT, group = Explant, color = Rat), linetype = 1, alpha = 1/3) +
 theme_classic ()


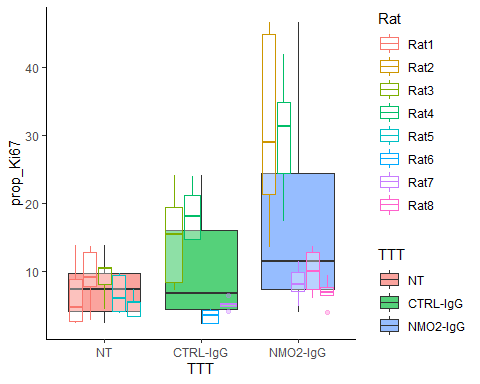


# modeles à effets mixtes
modmix7 <-lmerTest::lmer(prop_Ki67 ~ TTT + (1|Rat/Explant), data = data7)
summary(modmix7)

## Linear mixed model fit by REML. t-tests use Satterthwaite's method [
## lmerModLmerTest]
## Formula: prop_Ki67 ~ TTT + (1 | Rat/Explant)
## Data: data7
##
## REML criterion at convergence: 450.6
##
## Scaled residuals:
## Min 1Q Median 3Q Max
## -2.89756 -0.47009 -0.02225 0.30495 2.93184
##
## Random effects:
## Groups Name Variance Std.Dev.
## Explant:Rat (Intercept) 0.007537 0.08682
## Rat (Intercept) 67.773992 8.23250
## Residual 32.342610 5.68706
## Number of obs: 70, groups: Explant:Rat, 14; Rat, 8
##
## Fixed effects:
## Estimate Std. Error df t value Pr(>|t|)
## (Intercept) 5.933 3.771 10.414 1.573 0.1455
## TTTCTRL-IgG 5.430 3.215 6.449 1.689 0.1387
## TTTNMO2-IgG 12.866 3.820 7.192 3.368 0.0115 *
## ---
## Signif. codes: 0 '***' 0.001 '**' 0.01 '*' 0.05 '.' 0.1 ' ' 1
##
## Correlation of Fixed Effects:
## (Intr) TTTCTR
## TTTCTRL-IgG -0.559
## TTTNMO2-IgG -0.584 0.778

# vérifications des hypothéses : normalité des résidus et des effets aléatoires
qqPlot(resid(modmix7))


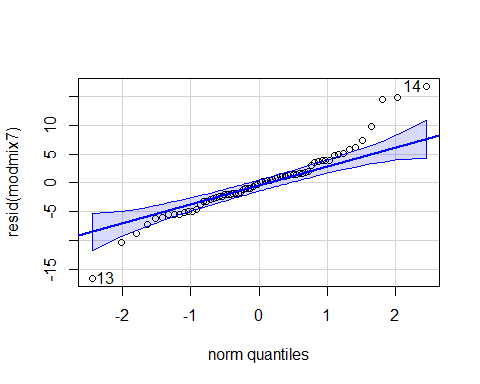


## [1] 14 13

qqPlot(ranef(modmix7)$"Explant:Rat"[,1])


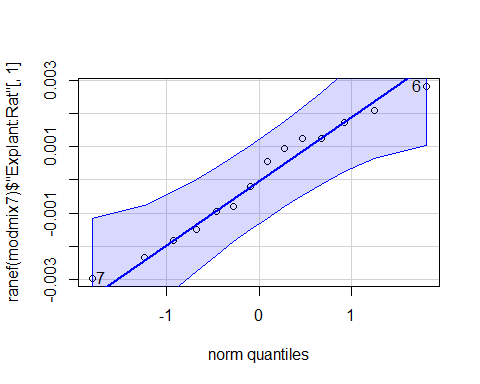


## [1] 7 6

qqPlot(ranef(modmix7)$`Rat`[,1])


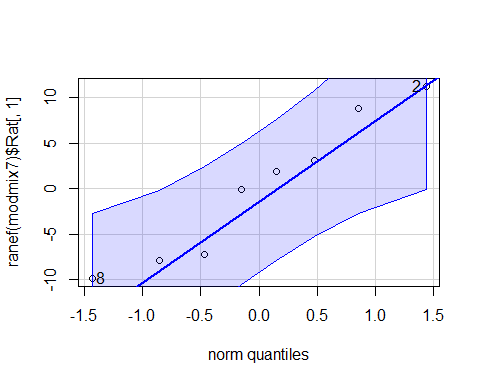


## [1] 2 8

#### Mean BBPA deviation

# import et preparation des donnÃ©es
data8 <- read.table("Mean_BBPA.csv", h = T, sep = ",")
data8$TTT <- relevel(as.factor(data8$TTT), "NT")
data8$Explant <- as.factor(data8$Explant)
data8$Rat <- as.factor(data8$Rat)

# representation graphique
ggplot(data8) +
 geom_boxplot (aes(y = Mean_BBPA, x = TTT, fill = TTT), alpha = 2/3) +
 geom_boxplot (aes(y = Mean_BBPA, x = TTT, group = Explant, color = Rat), linetype = 1, alpha = 1/3) +
 theme_classic ()


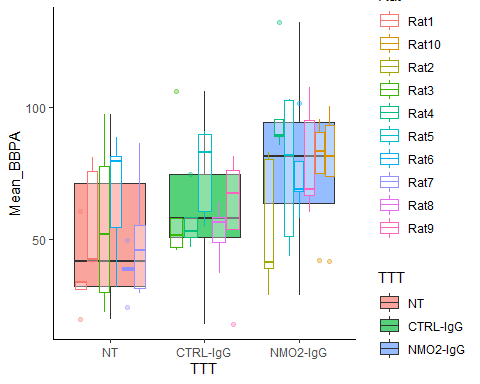


# modeles à effets mixtes
modmix8 <-lmerTest::lmer(Mean_BBPA ~ TTT + (1|Rat/Explant), data = data8)

## boundary (singular) fit: see ?isSingular

summary(modmix8)

## Linear mixed model fit by REML. t-tests use Satterthwaite's method [
## lmerModLmerTest]
## Formula: Mean_BBPA ~ TTT + (1 | Rat/Explant)
## Data: data8
##
## REML criterion at convergence: 784.9
##
## Scaled residuals:
## Min 1Q Median 3Q Max
## -2.0233 -0.5528 -0.1058 0.7294 2.2757
##
## Random effects:
## Groups Name Variance Std.Dev.
## Explant:Rat (Intercept) 43.72 6.612
## Rat (Intercept) 0.00 0.000
## Residual 446.72 21.136
## Number of obs: 89, groups: Explant:Rat, 18; Rat, 10
##
## Fixed effects:
## Estimate Std. Error df t value Pr(>|t|)
## (Intercept) 50.444 4.767 15.582 10.583 1.62e-08 ***
## TTTCTRL-IgG 10.946 7.024 15.229 1.558 0.139671
## TTTNMO2-IgG 26.737 6.460 15.283 4.139 0.000843 ***
## ---
## Signif. codes: 0 '***' 0.001 '**' 0.01 '*' 0.05 '.' 0.1 ' ' 1
##
## Correlation of Fixed Effects:
## (Intr) TTTCTR
## TTTCTRL-IgG -0.679
## TTTNMO2-IgG -0.738 0.501
## optimizer (nloptwrap) convergence code: 0 (OK)
## boundary (singular) fit: see ?isSingular

# vérifications des hypothéses : normalité des résidus et des effets aléatoires
qqPlot(resid(modmix8))


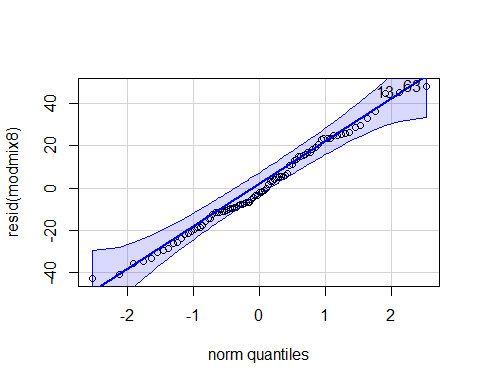


## [1] 63 13

qqPlot(ranef(modmix8)$"Explant:Rat"[,1])


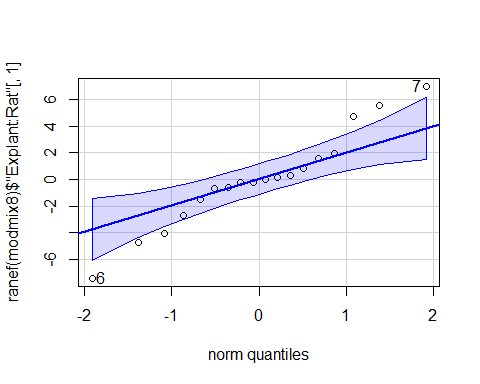


## [1] 6 7

qqPlot(ranef(modmix8)$`Rat`[,1])


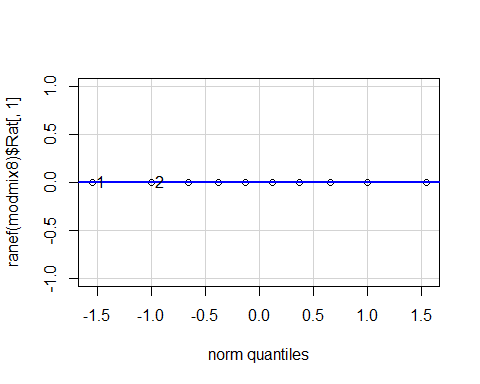


## [1] 1 2

Summary mixed models

summary(modmix1)

## Linear mixed model fit by REML. t-tests use Satterthwaite's method [
## lmerModLmerTest]
## Formula: Speed ~ TTT + (1 | Rat/Explant)
## Data: data
##
## REML criterion at convergence: 7474.5
##
## Scaled residuals:
## Min 1Q Median 3Q Max
## -2.8111 -0.5725 -0.0291 0.5218 6.0317
##
## Random effects:
## Groups Name Variance Std.Dev.
## Explant:Rat (Intercept) 2165 46.53
## Rat (Intercept) 1554 39.42
## Residual 1710 41.36
## Number of obs: 720, groups: Explant:Rat, 24; Rat, 14
##
## Fixed effects:
## Estimate Std. Error df t value Pr(>|t|)
## (Intercept) 265.102 25.075 14.041 10.573 4.54e-08 ***
## TTTCTRL-IgG 8.145 34.791 13.589 0.234 0.818
## TTTNMO2-IgG -22.704 32.328 18.036 -0.702 0.491
## ---
## Signif. codes: 0 '***' 0.001 '**' 0.01 '*' 0.05 '.' 0.1 ' ' 1
##
## Correlation of Fixed Effects:
## (Intr) TTTCTR
## TTTCTRL-IgG -0.717
## TTTNMO2-IgG -0.725 0.554

summary(modmix2)

## Linear mixed model fit by REML. t-tests use Satterthwaite's method [
## lmerModLmerTest]
## Formula: Directionality ~ TTT + (1 | Rat/Explant)
## Data: data2
##
## REML criterion at convergence: -2743.3
##
## Scaled residuals:
## Min 1Q Median 3Q Max
## -9.7196 -0.3921 0.0786 0.5002 3.1829
##
## Random effects:
## Groups Name Variance Std.Dev.
## Explant:Rat (Intercept) 7.847e-04 2.801e-02
## Rat (Intercept) 2.607e-18 1.615e-09
## Residual 1.140e-03 3.376e-02
## Number of obs: 720, groups: Explant:Rat, 24; Rat, 14
##
## Fixed effects:
## Estimate Std. Error df t value Pr(>|t|)
## (Intercept) 0.92351 0.01084 21.00000 85.186 <2e-16 ***
## TTTCTRL-IgG -0.00924 0.01485 21.00000 -0.622 0.540
## TTTNMO2-IgG -0.01311 0.01446 21.00000 -0.907 0.375
## ---
## Signif. codes: 0 '***' 0.001 '**' 0.01 '*' 0.05 '.' 0.1 ' ' 1
##
## Correlation of Fixed Effects:
## (Intr) TTTCTR
## TTTCTRL-IgG -0.730
## TTTNMO2-IgG -0.750 0.548
## optimizer (nloptwrap) convergence code: 0 (OK)
## boundary (singular) fit: see ?isSingular

summary(modmix3)

## Linear mixed model fit by REML. t-tests use Satterthwaite's method [
## lmerModLmerTest]
## Formula: X.agglo ~ TTT + Culture + (1 | Puit)
## Data: data3
##
## REML criterion at convergence: 2882.2
##
## Scaled residuals:
## Min 1Q Median 3Q Max
## -4.3640 -0.3085 -0.0102 0.4074 2.6528
##
## Random effects:
## Groups Name Variance Std.Dev.
## Puit (Intercept) 207.5 14.40
## Residual 271.8 16.49
## Number of obs: 343, groups: Puit, 37
##
## Fixed effects:
## Estimate Std. Error df t value Pr(>|t|)
## (Intercept) 28.705 8.653 26.182 3.317 0.002673 **
## TTTCTRL-IgG 5.196 11.007 28.237 0.472 0.640491
## TTTNMO1-IgG -7.103 10.042 26.054 -0.707 0.485610
## TTTNMO2-IgG 85.674 10.042 26.054 8.532 5.09e-09 ***
## TTTNMO3-IgG 16.153 10.623 25.356 1.521 0.140751
## TTTNMO4-IgG 44.247 10.623 25.356 4.165 0.000316 ***
## TTTNMO5-IgG 24.490 11.779 25.688 2.079 0.047735 *
## TTTNMO6-IgG 21.732 11.779 25.688 1.845 0.076596 .
## TTTNMO7-IgG 57.801 10.722 25.804 5.391 1.23e-05 ***
## CultureCE15 -10.070 7.506 23.774 -1.342 0.192413
## CultureCE6 -20.237 8.420 24.677 -2.404 0.024085 *
## CultureCE7 -27.849 8.166 26.407 -3.410 0.002099 **
## CultureCE8 -16.101 7.890 23.741 -2.041 0.052546 .
## ---
## Signif. codes: 0 '***' 0.001 '**' 0.01 '*' 0.05 '.' 0.1 ' ' 1

##
## Correlation matrix not shown by default, as p = 13 > 12.
## Use print(x, correlation=TRUE) or
## vcov(x) if you need it

summary(modmix4)

## Linear mixed model fit by REML. t-tests use Satterthwaite's method [
## lmerModLmerTest]
## Formula: Number.of.B.cells ~ TTT + (1 | Rat/Explant)
## Data: data4
##
## REML criterion at convergence: 729.6
##
## Scaled residuals:
## Min 1Q Median 3Q Max
## -2.46668 -0.48200 -0.08227 0.50170 2.58433
##
## Random effects:
## Groups Name Variance Std.Dev.
## Explant:Rat (Intercept) 84.73 9.205
## Rat (Intercept) 312.29 17.672
## Residual 154.24 12.420
## Number of obs: 90, groups: Explant:Rat, 18; Rat, 10
##
## Fixed effects:
## Estimate Std. Error df t value Pr(>|t|)
## (Intercept) 31.509 8.669 14.268 3.635 0.00263 **
## TTTCTRL-IgG 18.074 9.690 11.283 1.865 0.08836 .
## TTTNMO2-IgG 31.824 9.495 11.989 3.352 0.00577 **
## ---
## Signif. codes: 0 '***' 0.001 '**' 0.01 '*' 0.05 '.' 0.1 ' ' 1
##
## Correlation of Fixed Effects:
## (Intr) TTTCTR
## TTTCTRL-IgG -0.627
## TTTNMO2-IgG -0.661 0.685

summary(modmix5)

## Linear mixed model fit by REML. t-tests use Satterthwaite's method [
## lmerModLmerTest]
## Formula: Area ~ TTT + (1 | Rat/Explant)
## Data: data5
##
## REML criterion at convergence: 14108.3
##
## Scaled residuals:
## Min 1Q Median 3Q Max
## -3.0094 -0.6094 -0.0764 0.5069 10.3610
##
## Random effects:
## Groups Name Variance Std.Dev.
## Explant:Rat (Intercept) 272.1 16.49
## Rat (Intercept) 0.0 0.00
## Residual 1039.0 32.23
## Number of obs: 1440, groups: Explant:Rat, 12; Rat, 6
##
## Fixed effects:
## Estimate Std. Error df t value Pr(>|t|)
## (Intercept) 99.8168 8.4276 9.1570 11.844 7.38e-07 ***
## TTTCTRL-IgG -0.1508 12.8072 8.9736 -0.012 0.99086
## TTTNMO2-IgG 42.9102 11.2977 9.1275 3.798 0.00412 **
## ---
## Signif. codes: 0 '***' 0.001 '**' 0.01 '*' 0.05 '.' 0.1 ' ' 1
##
## Correlation of Fixed Effects:
## (Intr) TTTCTR
## TTTCTRL-IgG -0.658
## TTTNMO2-IgG -0.746 0.491
## optimizer (nloptwrap) convergence code: 0 (OK)
## boundary (singular) fit: see ?isSingular

summary(modmix6)

## Linear mixed model fit by REML. t-tests use Satterthwaite's method [
## lmerModLmerTest]
## Formula: Average_particle_size ~ TTT + Culture + (1 | Puit)
## Data: data6
##
## REML criterion at convergence: -376
##
## Scaled residuals:
## Min 1Q Median 3Q Max
## -2.5502 -0.5614 -0.0674 0.4293 5.0427
##
## Random effects:
## Groups Name Variance Std.Dev.
## Puit (Intercept) 0.003045 0.05519
## Residual 0.008867 0.09417
## Number of obs: 240, groups: Puit, 24
##
## Fixed effects:
## Estimate Std. Error df t value Pr(>|t|)
## (Intercept) 0.24285 0.04037 13.00000 6.016 4.33e-05 ***
## TTTCTRL-IgG 0.10794 0.05801 13.00000 1.861 0.08557 .
## TTTNMO1-IgG 0.01427 0.05120 13.00000 0.279 0.78490
## TTTNMO2-IgG 0.12983 0.05120 13.00000 2.536 0.02485 *
## TTTNMO3-IgG 0.12723 0.05120 13.00000 2.485 0.02735 *
## TTTNMO4-IgG 0.10657 0.05120 13.00000 2.081 0.05773 .
## TTTNMO5-IgG 0.04129 0.05809 13.00000 0.711 0.48975
## TTTNMO6-IgG 0.02114 0.05809 13.00000 0.364 0.72176
## TTTNMO7-IgG 0.06360 0.05120 13.00000 1.242 0.23611
## CultureCE15 0.03842 0.03093 13.00000 1.242 0.23604
## CultureCE8 -0.11217 0.03260 13.00000 -3.441 0.00439 **
## ---
## Signif. codes: 0 '***' 0.001 '**' 0.01 '*' 0.05 '.' 0.1 ' ' 1
##
## Correlation of Fixed Effects:
## (Intr) TTTCTR TTTNMO1 TTTNMO2 TTTNMO3 TTTNMO4 TTTNMO5 TTTNMO6
## TTTCTRL-IgG -0.590
## TTTNMO1-IgG -0.634 0.441
## TTTNMO2-IgG -0.634 0.441 0.500
## TTTNMO3-IgG -0.634 0.441 0.500 0.500
## TTTNMO4-IgG -0.634 0.441 0.500 0.500 0.500
## TTTNMO5-IgG -0.597 0.373 0.441 0.441 0.441 0.441
## TTTNMO6-IgG -0.597 0.373 0.441 0.441 0.441 0.441 0.417
## TTTNMO7-IgG -0.634 0.441 0.500 0.500 0.500 0.500 0.441 0.441
## CultureCE15 -0.369 0.138 0.000 0.000 0.000 0.000 -0.010 -0.010
## CultureCE8 -0.377 -0.019 0.000 0.000 0.000 0.000 0.150 0.150
## TTTNMO7 ClCE15
## TTTCTRL-IgG
## TTTNMO1-IgG
## TTTNMO2-IgG
## TTTNMO3-IgG
## TTTNMO4-IgG
## TTTNMO5-IgG
## TTTNMO6-IgG
## TTTNMO7-IgG
## CultureCE15 0.000
## CultureCE8 0.000 0.422

summary(modmix7)

## Linear mixed model fit by REML. t-tests use Satterthwaite's method [
## lmerModLmerTest]
## Formula: prop_Ki67 ~ TTT + (1 | Rat/Explant)
## Data: data7
##
## REML criterion at convergence: 450.6
##
## Scaled residuals:
## Min 1Q Median 3Q Max
## -2.89756 -0.47009 -0.02225 0.30495 2.93184
##
## Random effects:
## Groups Name Variance Std.Dev.
## Explant:Rat (Intercept) 0.007537 0.08682
## Rat (Intercept) 67.773992 8.23250
## Residual 32.342610 5.68706
## Number of obs: 70, groups: Explant:Rat, 14; Rat, 8
##
## Fixed effects:
## Estimate Std. Error df t value Pr(>|t|)
## (Intercept) 5.933 3.771 10.414 1.573 0.1455
## TTTCTRL-IgG 5.430 3.215 6.449 1.689 0.1387
## TTTNMO2-IgG 12.866 3.820 7.192 3.368 0.0115 *
## ---
## Signif. codes: 0 '***' 0.001 '**' 0.01 '*' 0.05 '.' 0.1 ' ' 1
##
## Correlation of Fixed Effects:
## (Intr) TTTCTR
## TTTCTRL-IgG -0.559
## TTTNMO2-IgG -0.584 0.778

summary(modmix8)

## Linear mixed model fit by REML. t-tests use Satterthwaite's method [
## lmerModLmerTest]
## Formula: Mean_BBPA ~ TTT + (1 | Rat/Explant)
## Data: data8
##
## REML criterion at convergence: 784.9
##
## Scaled residuals:
## Min 1Q Median 3Q Max
## -2.0233 -0.5528 -0.1058 0.7294 2.2757
##
## Random effects:
## Groups Name Variance Std.Dev.
## Explant:Rat (Intercept) 43.72 6.612
## Rat (Intercept) 0.00 0.000
## Residual 446.72 21.136
## Number of obs: 89, groups: Explant:Rat, 18; Rat, 10
##
## Fixed effects:
## Estimate Std. Error df t value Pr(>|t|)
## (Intercept) 50.444 4.767 15.582 10.583 1.62e-08 ***
## TTTCTRL-IgG 10.946 7.024 15.229 1.558 0.139671
## TTTNMO2-IgG 26.737 6.460 15.283 4.139 0.000843 ***
## ---
## Signif. codes: 0 '***' 0.001 '**' 0.01 '*' 0.05 '.' 0.1 ' ' 1
##
## Correlation of Fixed Effects:
## (Intr) TTTCTR
## TTTCTRL-IgG -0.679
## TTTNMO2-IgG -0.738 0.501
## optimizer (nloptwrap) convergence code: 0 (OK)
## boundary (singular) fit: see ?isSingular
